# Rab family of small GTPases: an updated view on their regulation and functions

**DOI:** 10.1111/febs.15453

**Published:** 2020-07-01

**Authors:** Yuta Homma, Shu Hiragi, Mitsunori Fukuda

**Affiliations:** ^1^ Laboratory of Membrane Trafficking Mechanisms Department of Integrative Life Sciences Graduate School of Life Sciences Tohoku University Sendai Japan

**Keywords:** effector, GAP, GEF, knockout, membrane traffic, organelle, post‐translational modification, Rab small GTPases

## Abstract

The Rab family of small GTPases regulates intracellular membrane trafficking by orchestrating the biogenesis, transport, tethering, and fusion of membrane‐bound organelles and vesicles. Like other small GTPases, Rabs cycle between two states, an active (GTP‐loaded) state and an inactive (GDP‐loaded) state, and their cycling is catalyzed by guanine nucleotide exchange factors (GEFs) and GTPase‐activating proteins (GAPs). Because an active form of each Rab localizes on a specific organelle (or vesicle) and recruits various effector proteins to facilitate each step of membrane trafficking, knowing when and where Rabs are activated and what effectors Rabs recruit is crucial to understand their functions. Since the discovery of Rabs, they have been regarded as one of the central hubs for membrane trafficking, and numerous biochemical and genetic studies have revealed the mechanisms of Rab functions in recent years. The results of these studies have included the identification and characterization of novel GEFs, GAPs, and effectors, as well as post‐translational modifications, for example, phosphorylation, of Rabs. Rab functions beyond the simple effector‐recruiting model are also emerging. Furthermore, the recently developed CRISPR/Cas technology has enabled acceleration of knockout analyses in both animals and cultured cells and revealed previously unknown physiological roles of many Rabs. In this review article, we provide the most up‐to‐date and comprehensive lists of GEFs, GAPs, effectors, and knockout phenotypes of mammalian Rabs and discuss recent findings in regard to their regulation and functions.

## Graphical abstract

The Rab family of small GTPases regulates intracellular membrane trafficking by localizing on specific organelles (or vesicles) and recruiting various effector proteins to facilitate each step of membrane trafficking. In this review article, we provide the most up‐to‐date and comprehensive lists of guanine nucleotide exchange factors, GTPase‐activating proteins, effectors, and knockout phenotypes of mammalian Rabs and discuss recent findings in regard to their regulation and functions.

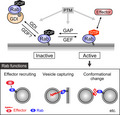

**Abbreviations**
DENN: differentially expressed in normal and neoplastic cells
EGF: epidermal growth factor
EGFR: epidermal growth factor receptor
ER: endoplasmic reticulum
EV: extracellular vesicle
GAP: GTPase‐activating protein
GDF: GDI displacement factor
GDI: GDP dissociation inhibitor
GEF: guanine nucleotide exchange factor
Hh: Hedgehog
HPS: Hermansky–Pudlak syndrome
IMPC: International Mouse Phenotyping Consortium
KO: knockout
LCV: *Legionella*‐containing vacuole
LRO: lysosome‐related organelle
LRRK1/2: leucine‐rich repeat kinase 1/2
MDCK: Madin–Darby canine kidney
NCBI: National Center for Biotechnology Information
PD: Parkinson's disease
PTM: post‐translational modification
REP: Rab escort protein
RILPL1/2: Rab interacting lysosomal protein‐like 1/2
sgRNA: single‐guide RNA
TBC: Tre‐2/Bub2/Cdc16
TBK1: TANK‐binding kinase 1
TGN: *trans*‐Golgi network
Vps: vacuolar protein sorting

## Introduction

**Fig. 1:**
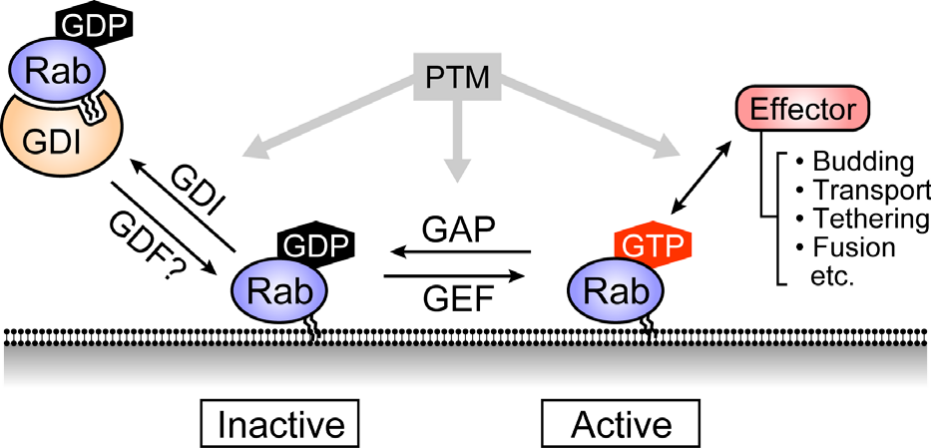
The Rab GTPase cycle. Rab GTPases are activated (GTP‐loaded) by guanine nucleotide exchange factors (GEFs) and inactivated (GDP‐loaded) by GTPase‐activating proteins (GAPs). Inactive Rabs bind to GDP dissociation inhibitor (GDI) and are retained in the cytosol [136]. GDI is thought to be dissociated by GDI displacement factor (GDF) [137], but whether this mechanism is applicable to all Rabs remains unclear. Active Rabs are associated with intracellular membranes and recruit specific effector proteins that regulate various steps of membrane trafficking, including budding, transport, tethering, and fusion of vesicles and organelles. Post‐translational modifications (PTMs), such as phosphorylation, of Rabs are thought to regulate their interaction with GDI, GEFs/GAPs, and effectors [138].

Eukaryotic cells possess a highly organized endomembrane system that compartmentalizes various biochemical reactions and enable the exchange of numerous molecules with the extracellular milieu by means of exocytosis and endocytosis. Many steps of membrane trafficking, including the biogenesis, transport, tethering, and fusion of membrane‐bound organelles and vesicles, are thought to be regulated by the Rab family of small GTPases [1, 2, 3, 4]. Like other small GTPases, Rabs cycle between two states, an active (GTP‐loaded) state and an inactive (GDP‐loaded) state, and the cycling is catalyzed by guanine nucleotide exchange factors (GEFs) and GTPase‐activating proteins (GAPs) (Fig. 1). Switch regions (switch I and II) of Rabs are known to undergo large conformational changes upon binding of GDP or GTP. When activated by a GEF, Rabs localize via their prenylated (or doubly prenylated) C termini to specific membranes of several compartments, such as the endoplasmic reticulum (ER), Golgi apparatus, secretory vesicles, endosomes, or lysosomes, where they recruit effector proteins (i.e., active Rab‐binding partners) that regulate different steps of membrane trafficking. Consequently, knowing when and where Rabs are activated and what kinds of effectors they recruit are crucial to achieving a complete understanding of Rab functions during membrane trafficking. In this review article, we describe recent findings in regard to the regulation and functions of Rabs, and we update the lists of GEFs, GAPs, effectors, and knockout (KO) phenotypes of mammalian Rabs.

## Nomenclature of mammalian *Rab* genes

**Fig. 2:**
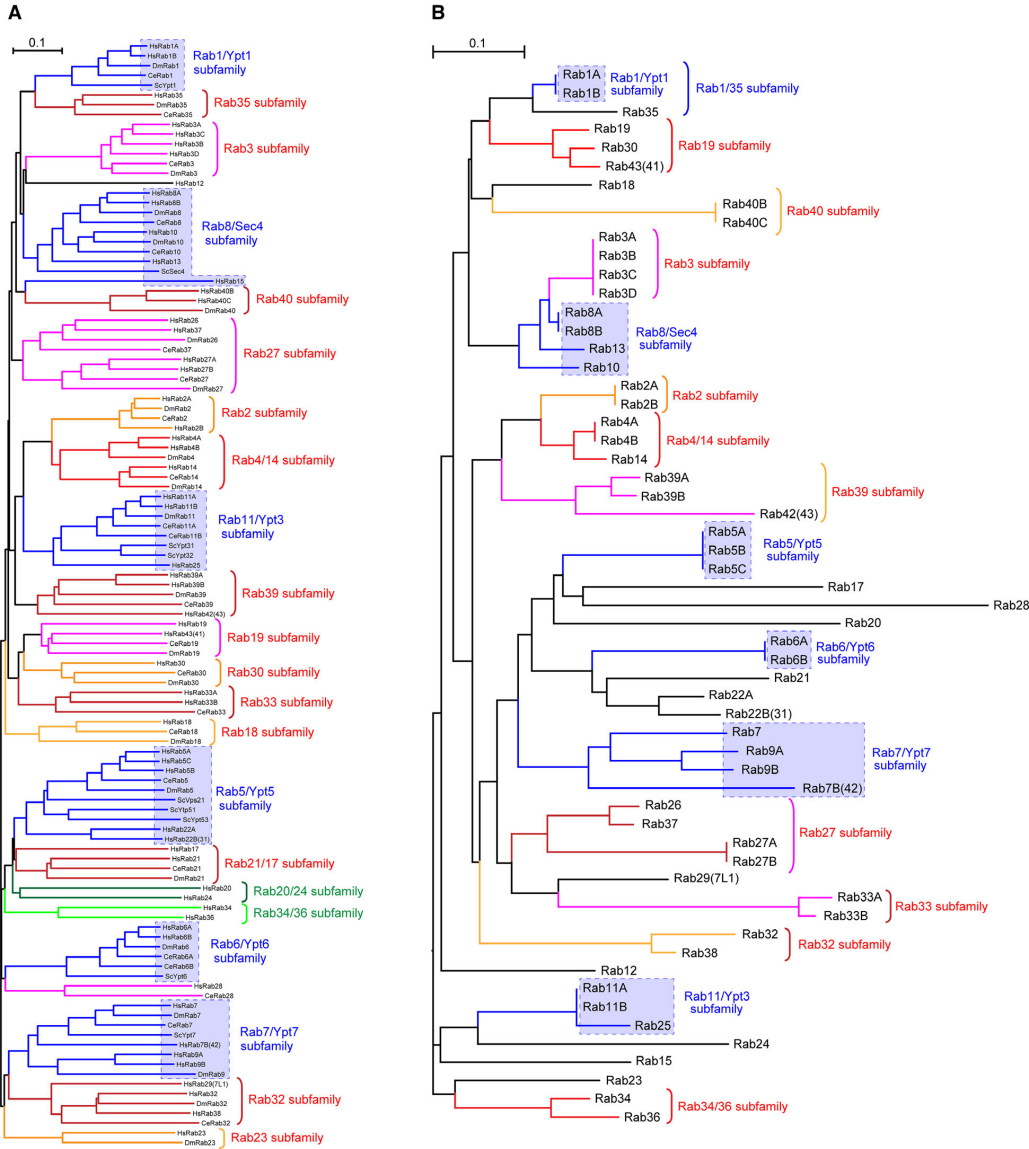
Phylogenetic analysis of Rabs. Amino acid sequences of the full length (A) or the switch II region (B) of human Rabs have been aligned using the clustalw software program (version 2.1; available at http://clustalw.ddbj.nig.ac.jp/top‐e.html) set at the default parameters and their phylogenetic tree was drawn by the neighbor‐joining method. All Rab sequences used for the phylogenetic analysis were obtained from the NCBI database (see also Figs S1 and S2). Note that the classifications of subfamilies are similar to each other and to the classification based on the full‐length sequences of more than 7600 Rabs from various species [8]. The Rab subfamily members that have been conserved from budding yeasts to humans are enclosed in boxes composed of dashed blue lines. (A) includes budding yeast (Sc) Ypts, *Caenorhabditis elegans* (Ce) Rabs, and *Drosophila melanogaster* (Dm) Rabs that are also conserved in humans. Human‐ or primate‐specific Rabs, that is, Rab6C, Rab41/6D, Rab40A, and Rab40AL, have been excluded from this figure.

Typical *Rab* genes encode a small GTPase consisting of around 200–250 amino acids, and approximately 60 *Rab* genes have been identified in mammals to date (Table 1) [5, 6, 7, 8]. The Rab names used in this review are according to the National Center for Biotechnology Information (NCBI) gene symbols; however, the numbering of some Rabs differs among studies (indicated in parentheses in Table 1), and the current Rab names do not always reflect their sequence similarities. A phylogenetic analysis actually suggested that the following Rabs are classified into the same subfamilies (i.e., paralogs) [7, 8]: Rab11 and Rab25; Rab19 and Rab43; Rab22A and Rab31/22B; Rab26 and Rab37; Rab32 and Rab38; Rab34 and Rab36; and Rab39 and Rab42. This classification is well consistent with a classification based on alignments of the switch II region sequences alone (Fig. 2), indicating that the switch II region is the main determinant of not only Rab effector binding specificity [9] but Rab identity as well. The functional relationships between these paralogous Rabs remain unclear and would be worth investigating in the future.

**Table 1:** Mouse and human *Rab* genes. The nomenclature of Rabs in this review is according to the NCBI database. The names of several Rabs in the report by Itoh *et al*. [6] are different (indicated in parentheses). Representative phenotypes of mutant animals are shown in this table, and all other phenotypes together with their respective references (PubMed ID) are listed in Table S2. The phenotypes of several knockout mice (indicated by asterisks) are available from at the following URL: https://www.mousephenotype.org/. Rab33A (Cat#: RBRC05799) and Rab33B KO mice (Cat#: RBRC05800) (indicated by double asterisks) are also available from the RIKEN BioResource Research Center (https://mus.brc.riken.jp/en/). AJ, adherens junction; AP, autophagosome; C, cilium; E, endosome; EE, early endosome; ERC, endocytic recycling compartment; G, Golgi; KO, knockout; LE, late endosome; LRO, lysosome‐related organelle; LD, lipid droplet; LY, lysosome; MS, melanosome; MT, mitochondrion; N, nucleus; PM, plasma membrane; P, peroxisome; PS, phagosome; RE, recycling endosome; SG, secretory granule; SV, synaptic vesicle; TE, tubular endosome; TGN, *trans*‐Golgi network; TJ, tight junction.

Name (NCBI)	Gene ID		Disease and Mutant animal	Knockout phenotype	Subcellular localization
	Human	Mouse			
Rab1A	5861	19324			EE/ER/G/MS
Rab1B	81876	76308			ER/G
Rab2A	5862	59021		Preweaning lethality*	G/AP
Rab2B	84932	76338			G/AP
Rab3A	5864	19339	*Earlybird* [139]	Viable and fertile; perinatal lethality of Rab3A/B/C/D quadruple KO mice; 30% reduction of Ca^2+^‐triggered synaptic release [140, 141, 142]	Acrosome/MS/SG/SV
Rab3B	5865	69908		Viable and fertile [140]	G/RE/SG/SV/TJ
Rab3C	115827	67295		Viable and fertile [140]	RE/SG/SV
Rab3D	9545	19340		Viable and fertile [140]	G/LRO/SG/SV/TGN
Rab4A	5867	19341			EE/RE
Rab4B	53916	19342		Adipocyte hypertrophy and insulin resistance in T cell‐specific KO mice [143]	EE/RE
Rab5A	5868	271457			EE/early PS
Rab5B	5869	19344			EE/PM
Rab5C	5878	19345		Preweaning lethality*	EE/early PS
Rab6A	5870	19346		Embryonic lethal; defects in basement membrane formation in KO embryos [144, 145]	G/P/TGN‐derived vesicle
Rab6B	51560	270192			G
Rab6C	84084	–			
Rab41 (6D)	347517	–			ER/G
Rab7A (7)	7879	19349	Charcot–Marie–Tooth type 2B [146]	Embryonic lethal; defects in microautophagy in the visceral endoderm of KO embryos [147]	AP/LE/LY/PS
Rab7B (42)	338382	226421			LE
Rab8A	4218	17274	Related to microvillus inclusion disease [148]	Die at postnatal week 4, defects in apical protein localization, and microvillus inclusion bodies [130, 148, 149]	C/RE/SG/TE/TGN
Rab8B	51762	235442		No obvious phenotype; Rab8A/B double KO mice die at postnatal week 3 [130]	AJ/TGN
Rab9A	9367	56382			LE/TGN
Rab9B	51209	319642			LE
Rab10	10890	19325		Embryonic lethal [120]	E/ER/P/RE/TE/TGN
Rab11A	8766	53869		Embryonic lethal [150, 151, 152]	RE/TGN/TE/TGN‐derived vesicle
Rab11B	9230	19326	Intellectual disability [153]		RE
Rab12	201475	19328			G/SG/RE/LE/LY
Rab13	5872	68328		Viable; reduced lymphocyte numbers and reduced lymophocyte trafficking [121]	RE/TE/TGN/TJ
Rab14	51552	68365			EE/G/P/PS/TGN
Rab15	376267	104886			EE/RE
Rab17	64284	19329			RE/MS
Rab18	22931	19330	Warburg Micro syndrome [154]	Viable and fertile; ocular and neurological abnormalities [155]	ER/G/LD/P/SG
Rab19	401409	19331			G
Rab20	55647	19332		Decreased formation of *Mycobacterium tuberculosis*‐containing proteolytic phagosomes [119]	ER/G/LE/PS
Rab21	23011	216344		Preweaning lethality*	EE
Rab22A	57403	19334			EE/RE/PS/TE
Rab22B (31)	11031	106572			G/TGN/PS
Rab23	51715	19335	Carpenter syndrome [156], *open brain* [157]	Embryonic lethality; neural‐tube defects [157]	GAS‐containing AP/E/PM/C
Rab24	53917	19336	Canine hereditary ataxia [158]		AP/ER/G/LE/N
Rab25	57111	53868		Increased tumor formation [159, 160]	RE
Rab26	25837	328778		Decreased microvascular barrier function [161]	AP/LY/SG
Rab27A	5873	11891	Griscelli syndrome type 2 [162], *ashen* [163]	Hypopigmentation; immunodeficincy [164, 165, 166]	MS/LRO/SG
Rab27B	5874	80718		Secretory defects in various endocrine, exocrine, and immune cells [167, 168, 169]	MS/LRO/SG/SV
Rab28	9364	100972	Cone‐rod dystrophy [170]	Retina degeneration [171]	
Rab29 (7L1)	8934	226422	Association with Parkinson's disease [172]	Renal enlargement and discoloration [173]	G/RE/TGN
Rab30	27314	75985			G
Rab32	10981	67844		Increased susceptibility to *Salmonella* Typhi [117]; coat and eye pigment dilution, some enlarged lung multilamellar bodies, and prolonged bleeding in Rab32/38 DKO mice [114]	ER/G/LRO/MS/MT
Rab33A	9363	19337		Viable and fertile**	G/SG/SV precursor
Rab33B	83452	19338	Dyggve–Melchior–Clausen syndrome [174, 175]	Viable and fertile**	AP/G
Rab34	83871	19376		Preweaning lethality; polydactyly; cleft‐lip/palate; ciliogenesis defect [124, 125]	G/PS
Rab35	11021	77407	Somatic mutations in human tumors [176]	Preweaning lethality*	EE/PM/RE
Rab36	9609	76877			G/RE
Rab37	326624	58222			SG
Rab38	23682	72433	*chocolate* [177]*, Ruby* [178]	Hypopigmentation; prolonged bleeding; pulmonary fibrosis [179]; coat and eye pigment dilution, some enlarged lung multilamellar bodies, and prolonged bleeding in Rab32/38 DKO mice [114]	G/LRO/MS
Rab39A	54734	270160		Viable; reduced cross‐presentation by dendritic cells [122]	G/LE/PS
Rab39B	116442	67790	X‐linked mental retardation/Parkinson's disease [180, 181]		ER/G
Rab40A	142684	‐			
Rab40B	10966	217371			TGN‐derived vesicle
Rab40C	57799	224624		Subviable*	ERC/LD
Rab40AL	282808	–			G
Rab42 (43)	115273	242681			G
Rab43 (41)	339122	69834	Association with a hereditary liver–colon cancer syndrome [182]	Viable and fertile; reduced cross‐presentation by dendritic cells [183]	G

**Fig. 3:**
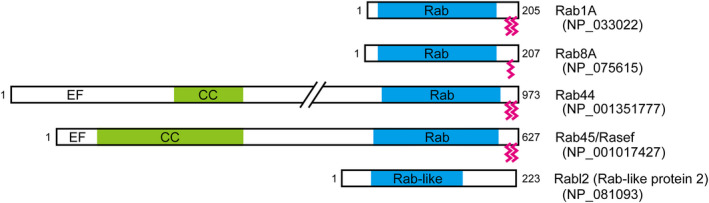
Typical Rabs and Rab‐related proteins. A comparison between typical Rabs (mouse Rab1A and Rab8A) and Rab‐related proteins (mouse Rab44, Rab45, and Rabl2) and their domain architecture are shown. The numbers in parentheses are the protein ID numbers of the respective proteins in the NCBI. Rab44 and Rab45 have long N‐terminal regions containing EF‐hand and coiled‐coil (CC) domains, in addition to their C‐terminal Rab‐like GTPase domains. Rabl2, an example of ‘Rab‐like’ proteins, has a Rab‐like GTPase domain but lacks a C‐terminal prenylation site (indicated by the pink lines), which is required for membrane insertion.

In addition to the above typical Rabs, there are several Rab‐related proteins (nevertheless referred to as Rabs by the NCBI) that fall outside the classical Rab category. Rab44, Rab45, and CRACR2A/Rab46 have long N‐terminal regions containing EF‐hand and coiled‐coil domains in addition to the C‐terminal Rab‐like GTPase domains (Fig. 3). The functions of these ‘large Rab GTPases’ have only recently begun to be investigated [10, 11, 12], and they are not discussed in this review. There are also six ‘Rab‐like’ proteins (Rabl2A, Rabl2B, Rabl3, Rabl4/Ift27, Rabl5/Ift22, and Rabl6), which contain only a Rab‐like GTPase domain, but they are not expected to regulate membrane trafficking, because they lack C‐terminal prenylation, which is required for membrane insertion. Actually, several Rab‐like proteins, such as Rabl4 and Rabl5, have been shown to regulate intraflagellar transport in cilia (i.e., the bidirectional transport of protein complexes required for cilium formation along axonemal microtubules) [13].

## Rab regulation

This section summarizes recent studies on newly discovered Rab regulation mechanisms and the life cycle of Rab proteins.

### mRNA compartmentalization

One member of the Rab family, Rab13, undergoes unique mRNA regulation. A genome‐wide microarray analysis revealed that *Rab13* mRNA is enriched in cell protrusions induced by migratory stimuli [14]. The 3′‐untranslated region of *Rab13* mRNA has been shown to be necessary and sufficient for transport to and accumulation at the tip of the protrusions in a microtubule‐dependent manner, which presumably facilitates local translation of Rab13. Moreover, *Rab13* mRNA has been found to be highly enriched (> 200‐fold) in extracellular vesicles (EVs) in comparison with the cell bodies of *KRAS*‐mutated colorectal cancer cells [15]. These EVs can be transferred to recipient cells and enable Rab13 translation within them. Because Rab13 has been implicated in cancer progression (reviewed in Ref. [16]), the relation between such mRNA regulation and cancer biology would be worth investigating in the future.

### Prenylation and activation

After Rab proteins are translated, they first bind to Rab escort protein (REP) and undergo geranylgeranyl transferase‐mediated prenylation at their C terminus, which enables them to be inserted into hydrophobic lipid bilayers (reviewed in Ref. [17]). Because inactive (GDP‐loaded) Rabs are extracted from membranes by GDP dissociation inhibitor (GDI), which masks their prenylated C terminus, Rabs need to be activated (GTP‐loaded) by GEFs to associate with specific membranes after release from GDI (Fig. 1). Consequently, Rab‐GEFs are thought to be major determinants of the specific Rab localizations to various organelles. For this reason, it is necessary to identify Rab‐GEFs in order to understand the spatiotemporal regulation of Rabs. To date, at least Drr1 (Rab1‐GEF), Rabex‐5 (Rab5‐GEF), Rabin8 (Rab8‐GEF), Rab3GAP1/2 (Rab18‐GEF), DENN/Rab3GEP (Rab27‐GEF), and HPS1/4 (Rab32/38‐GEF) have been shown to be necessary and/or sufficient for the proper localization of the corresponding Rabs [18, 19, 20, 21]. In addition, some Rab‐GEFs have been shown to also act as effectors of other Rabs (e.g., Rab8‐GEFs Rabin8 and GRAB act as Rab11 effectors). Such Rab‐GEFs mediate the transition of membrane (or organelle) identity from a ‘Rab A’‐positive membrane compartment to a ‘Rab B’‐positive membrane compartment by activating Rab B via a Rab A effector, which also functions as a Rab B‐GEF. These sequential activation mechanisms are called ‘Rab cascades’ (see reviews [22, 23] for details). The C‐terminal tail of yeast Ypt1 (mammalian Rab1 homolog) and of yeast Ypt31/32 (mammalian Rab11 homolog) has recently been shown to be critical for differential activation by the TRAPPIII complex and TRAPPII complex, respectively [24]. Remarkably, although these multisubunit complexes share the same catalytic subunits, the TRAPPII‐specific bulky subunits seem to keep the catalytic site of TRAPPII farther from the membrane surface than those of TRAPPIII. Thus, only Ypt31/32, which has a longer C‐terminal tail than Ypt1 does, is able to reach the catalytic site of TRAPPII, thereby ensuring specific activation of Ypt31/32, but not Ypt1, on the *trans*‐Golgi network (TGN). Mammalian TRAPPII/III complexes may also function as specific Rab‐GEFs, but their GEF activity and specificity remain to be determined.

The largest group of mammalian Rab‐GEFs is differentially expressed in normal and neoplastic cells (DENN) domain‐containing proteins [25], whose involvement in Rab regulation was first revealed by a genetic study using *C. elegans* [26]. Other Rab‐GEFs include vacuolar protein sorting 9 (Vps9) domain‐containing proteins, Sec2 domain‐containing proteins, and some heterodimeric or multimeric complexes (see reviews [27, 28] for details). Furthermore, the Fuzzy/Inturned complex and Plekhg5 have recently been added to the Rab‐GEF list as Rab23‐GEF and Rab26‐GEF, respectively [29, 30] (see Table S1).

### Phosphorylation

Phosphorylation is one of the most common post‐translational modifications (PTMs) of proteins. Previous studies on Rab phosphorylation are summarized in Table 2. The first report on Rab phosphorylation dates back to 1991, and it states that Rab1 and Rab4 are phosphorylated by a mitotic kinase CDK1, but that Rab2 or Rab6 is not [31]. In particular, almost the entire pool of Rab4 becomes phosphorylated during mitosis, thereby preventing it from associating with endosomal membranes [32]. These findings are consistent with the temporary cessation of membrane trafficking that occurs during mitosis. Rab7A has recently been shown to be phosphorylated under the following cellular conditions. Upon strong epidermal growth factor (EGF) stimulation, the activated epidermal growth factor receptors (EGFRs) are rapidly endocytosed and degraded in lysosomes. Phosphorylation of Rab7A at Y183 is induced during this event and is required for EGFRs to be efficiently delivered to lysosomes, rather than being recycled back to the plasma membrane [33]. Although the kinase responsible has yet to be identified, another report has stated that Src is capable of phosphorylating the Y183 of Rab7A [34]. On the other hand, Rab7A is phosphorylated at S72 by TANK‐binding kinase 1 (TBK1), a critical kinase for PINK1‐PARKIN‐mediated mitophagy [35]. Mitochondrial depolarization induces TBK1‐dependent phosphorylation of Rab7A, which leads to increased binding to its interactor folliculin [36], which is also required for mitophagy. The S72 of Rab7A is also phosphorylated by leucine‐rich repeat kinase 1 (LRRK1) [37], and both phosphorylated residues of Rab7A (S72 and Y183) have been shown to be dephosphorylated by PTEN [38]. Thus, the function of Rab7A is regulated by context‐dependent phosphorylation and dephosphorylation.

**Table 2:** Rab phosphorylation. Only studies that clarified the effect of phosphorylation on Rab function are shown. Because of space limitations, only the first two reports of Rab phosphorylation by LRRK2 (see text for details) [39, 40] are listed in this table.

Kinase	Phosphorylated Rab (residue)	Effect (−), inhibition; (+), promotion	Condition	Reference
CDK1	1A, 4A (S196)	(−) membrane association	During mitosis	[31, 32]
LRRK1	7A (S72)	(+) effector (RILP) binding	Expressing a hyperactive mutant of LRRK1 (Y944F)	[37]
LRRK2	3, 8, 10, 12, 29, 35, 43, etc. (T73 of Rab10 and the equivalent S/Ts of other Rabs)	(−) GDI binding (−) GEF (Rabin8) binding	Expressing a pathogenic mutant of LRRK2 (G2019S)	[39, 40]
PKC (α/β/γ)	6	(−) membrane association	Thrombin‐stimulated platelets	[184]
PKCα	37 (T172)	(−) GTP loading	Lung cancer cells	[185]
PKCα, PKCβII	11 (S177)	(−) TfR recycling	PMA treatment	[186]
PKCε	5A (T7)	(+) endosome trafficking for cell migration	LFA‐1/chemokine‐stimulated T cells	[187]
TAK1	1A (T75)	(−) GDI binding (+) Golgi localization	–	[188]
TBK1	7A (S72)	(−) GDI binding (+) effector (folliculin) binding	Upon mitochondrial depolarization	[35]
Src	7A (Y183)	(−) effector (RILP) binding	EGF treatment	[34]
Src	34 (Y247)	(+) β3‐integrin recycling for cell migration	During cell adhesion	[189]
–	7A (Y183)	(+) EGFR degradation	EGF treatment	[33]
–	8 (S111)	(−) GEF (Rabin8) binding	Upon mitochondrial depolarization	[50]

Rab phosphorylation has recently been receiving increasing attention, because LRRK2, a Parkinson's disease (PD)‐associated kinase, has been found to phosphorylate a subset of Rabs. A phosphoproteomic screen designed to identify physiological LRRK2 substrates identified Rab10 as the most promising candidate [39]. Its phosphorylation site (T73) is located within the conserved switch II region, and the equivalent Ser/Thr residue of other Rabs, including Rab3A/B/C/D, Rab8A/B, Rab12, Rab29, and Rab35, can also be phosphorylated by LRRK2 [40], while the phosphorylation sites of Rab8, Rab10, and Rab35 are dephosphorylated by PPM1H phosphatase [41]. Whereas the phosphorylation attenuates the interactions between these Rabs and GDI and several other binding partners, phosphorylated Rab8 and Rab10 strongly bind to Rab interacting lysosomal protein‐like 1 (RILPL1) and RILPL2. In particular, the phospho‐Rab10–RILPL1 interaction negatively regulates primary ciliogenesis, which may contribute to PD pathologies [42]. Rab phosphorylation by LRRK2 is also involved in lysosomal and centrosomal homeostasis and in Rab35‐mediated α‐synuclein propagation [43, 44, 45]. Another remarkable link between Rab and LRRK2 is that Rab29 (also known as Rab7L1), which is encoded within the PD‐linked locus (*PARK16*), directly binds to LRRK2 and recruits it to the Golgi apparatus, thereby greatly increasing its kinase activity [46, 47, 48, 49]. Moreover, another PD‐associated kinase PINK1 has been shown to induce Rab8 phosphorylation at S111 upon mitochondrial depolarization, which inhibits the interaction between Rab8 and its GEF, Rabin8 [50]. Taken together, the evidence increasingly indicates that phosphorylation is a crucial modulatory mechanism of Rab function in response to a variety of cellular events and that its dysregulation results in diseases such as PD.

### Other PTMs

Other known PTMs of Rab proteins include serotonylation, AMPylation, phosphocholination, palmitoylation, and ubiquitination. Non‐neuronal serotonin has been shown to be covalently bound to Rab4 (in platelets) and Rab3/27 (in β‐cells), which presumably contributes to the secretion of α‐granules and insulin, respectively [51, 52]. During *Legionella pneumophila* infection, Rab1 is AMPylated and phosphocholinated by bacterial proteins DrrA and AnkX, respectively [53, 54, 55, 56]. These modifications occur at the switch II region and affect the interaction of Rab1 with host proteins such as TBC1D20 (Rab1‐GAP), MICAL‐3 (Rab1 effector), and GDI. Rab35 is also phosphocholinated by AnkX and becomes incapable of being activated by its GEF DENND1A [54]. In addition, the palmitoylation of Rab7A at C83/C84 has been shown to be required for efficient interaction with the retromer complex and modulation of endosome‐to‐TGN trafficking of lysosomal sorting receptors [57].

While ubiquitination of transmembrane protein cargos is known to be the sorting signal for endocytic degradation [58], components of the membrane trafficking machinery themselves, including Rabs, are also regulated by ubiquitination. The first report on Rab ubiquitination stated that an E3 ligase, HACE1, ubiquitinates Rab11A at K145, which promotes activation of Rab11A and recycling of a β2‐adrenergic receptor [59]. Rab5A and Rab7A have also subsequently been shown to be ubiquitinated. Structural and biochemical analyses have revealed that ubiquitination of Rab5A impairs binding to its effector, EEA1, but the corresponding E3 ligase is unknown [60]. The K38 of Rab7A has been shown to be ubiquitinated by PARKIN and deubiquitinated by USP32 [61, 62]. Although the effect of this ubiquitination on effector binding is a matter of controversy, knockdown of either of these enzymes, as well as Rab7A [63, 64, 65], results in enlarged late endosomes (or lysosomes), suggesting that the ubiquitination/deubiquitination cycle is important for Rab7A function. Although ubiquitin is usually bound to a lysine residue of a specific substrate by the sequential action of E1, E2, and E3 enzymes, *Legionella* SidE family proteins atypically ubiquitinate a serine residue of Rab33B in a E1/E2‐independent manner [66, 67]. Determining whether and how this modification allows bacterial survival in host cells will require further investigation.

### Inactivation, stabilization, and degradation

Although Rab proteins themselves are GTPases that can hydrolyze their bound GTP to GDP, Rab‐GAPs enable more rapid and regulated inactivation of Rabs by enhancing their intrinsic GTPase activity. For example, TBC1D4 [also known as AS160 (Akt substrate of 160 kDa)] inactivates Rab8, Rab10, and Rab13, thereby preventing surface expression of GLUT4 in unstimulated skeletal muscles and adipocytes. Insulin stimulation activates these Rabs, because TBC1D4 phosphorylation by Akt attenuates its GAP activity, thus enabling insulin‐dependent GLUT4 translocation to the plasma membrane (reviewed in Ref. [68]). Most Rab‐GAPs identified to date are members of the Tre‐2/Bub2/Cdc16 (TBC) domain‐containing protein family and are encoded by ~ 40 genes in mammals [69] (see Table S1). Each TBC protein is thought to exert GAP activity on specific Rab family proteins, although their corresponding substrates have not been fully clarified [6]. Recent research on TBC family proteins has delineated the roles of TBC1D6 (Rab26‐GAP), TBC1D9B (Rab11‐GAP), TBC1D25/OATL1 (Rab33B‐GAP), and RUTBC1 (Rab32/38‐GAP) in GPCR trafficking, epithelial transcytosis, autophagosome maturation, and melanogenesis, respectively [70, 71, 72, 73, 74]. Moreover, TBC1D15 (Rab7A‐GAP) has been shown to be recruited to mitochondria by binding to Fis1 and to drive untethering of mitochondria–lysosome contacts by inactivating Rab7A [75, 76, 77]. Interestingly, expression of constitutively active Rab7A or GAP activity‐deficient mutants of TBC1D15 increases contact duration and inhibits mitochondrial fission events, suggesting that spatiotemporal regulation of Rab7A is important to the dynamics of mitochondria–lysosome contacts, which may determine the place and timing of mitochondrial fission. On the other hand, the GAP activity‐independent functions of several TBC family proteins, including TBC1D12, TBC1D14, TBC1D23, and TBC1D32/BROMI, have also been determined [78, 79, 80, 81]; for example, TBC1D23 is involved in endosomal vesicle capture at the *trans*‐Golgi in a GAP activity‐independent manner [80]. Future studies should provide a more comprehensive understanding of the roles of TBC family proteins and the regulation of Rab inactivation.

GDI extracts inactivated Rabs from membranes and masks their hydrophobic prenyl moiety, thereby retaining them in the cytosol for next use. In addition to this general mechanism, stabilization and degradation systems that are specific to a subset of Rabs have recently been reported. RABIF (also known as Mss4) acts as a holdase (ATP‐independent chaperone) of Rab8, Rab10, and Rab13, and its knockout leads to rapid proteasomal degradation of these Rabs [82]. Furthermore, hydrophobic residues within the switch I region of inactive Rab8 are recognized by BAG6, which targets Rab8 to proteasomes for degradation [83]. BAG6 also recognizes Rab10 and Rab13. These findings suggest that Rab8, Rab10, and Rab13 (yeast Sec4 homologs) are relatively unstable (especially in their inactive form) and/or prone to aggregate during folding and thus require specific quality control machineries to maintain their protein levels and prevent cytotoxic aggregation.

## New molecular mechanisms of Rab functions

Because the basic concept of the function of Rab proteins is that each family member decorates the surface of a specific organelle and recruits effectors that mediate membrane trafficking, effector identification is paramount to understanding the functions of each Rab. The first Rab effector was identified by a cross‐linking method, by using a membrane fraction from bovine brain and bacterially purified Rab3A loaded with GTPγS, a nonhydrolyzable GTP analog [84, 85]. GST pull‐down and yeast two‐hybrid screens that have used Rabs as bait have subsequently succeeded in identifying several effectors in early Rab studies [86, 87, 88, 89, 90]. Both methods are easy to perform and have continued to be used in recent works that have identified EHBP1L1 (a Rab8 effector for polarized transport) [91], Sec16A (a Rab10 effector for insulin secretion) [92], and RELCH (a Rab11 effector for cholesterol transport) [93]. They have also been applied to much larger scale analyses [94, 95, 96] that have provided clues to unveiling the roles of several Rabs. For example, Rab2 has been shown to interact with the HOPS complex (a tethering factor) and mediate autophagosome–lysosome fusion [97], while Rab18 has been demonstrated to bind to the NAG–RINT1–ZW10 complex (an interactor of ER‐associated Q‐SNAREs) and bridge ER–lipid droplet contacts [98].

Despite these efforts, there are still many Rabs to which few or no effectors have been assigned. One promising approach to further explore possibly weak and transient interactions between Rabs and their effectors is to use the recently developed proximity biotinylation techniques [99, 100]. Actually, APEX2 (an engineered form of a soybean ascorbate peroxidase)‐tagged Rab4, Rab5, Rab7A, and Rab21 have enabled identification and comparison of their interactors in living cells, and the results revealed that a Rab21–WASH complex interaction is required for trafficking of a subset of clathrin‐independent cargos [101]. However, two drawbacks of this method in terms of Rab‐effector identification need to be considered: (a) possible contamination by noninteractors that are merely localized on the same organelles, and (b) the difficulty of determining the nucleotide dependency of the interactions, because constitutively negative Rab mutants are often mislocalized or not membrane‐bound. The ‘mitoID’ method has recently been developed to overcome these drawbacks [102]. In brief, Rab (constitutively active/negative)‐BirA* (an *Escherichia coli* biotin ligase mutant) fusion proteins are ectopically targeted to mitochondria to eliminate background proteins identified in common. Many effectors of Rab2A, 6A, 7A, 8A, 9A, 10, 11A, 18, 30, and 33B, as well as several of their GEFs and GAPs, have been successfully identified by using this method. Although the mitoID method may be unsuited for effectors that require a specific lipid for membrane association [102], application to the other Rabs should be informative.

**Fig. 4:**
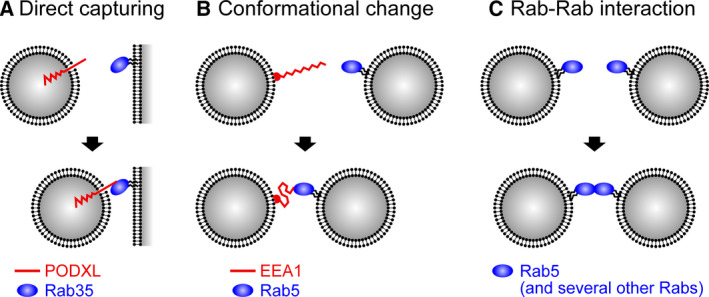
New mechanistic models of Rab‐mediated membrane tethering. (A) Podocalyxin (PODXL)‐containing vesicles are directly captured by Rab35 on the target membrane [105]. (B) Binding of EEA1 to Rab5 enables a conformational change in EEA1, which generates the pulling force that brings two endosomes together [106]. (C) Vesicle tethering can be induced by homo‐ and hetero‐typic interactions of Rabs in the absence of any effectors *in vitro* [109]. See the text for details.

Vesicle tethering is thought to be an important step in the proper recognition of target membranes and efficient fusion to them [103]. Whereas many Rabs facilitate this process by recruiting tethering factors, including golgins, COG complex, HOPS complex, and exocyst complex, new modes of Rab‐mediated tethering beyond such a simple ‘effector recruiting’ model have been reported (Fig. 4). During lumenogenesisof epithelial cell, vesicles containing apical membrane proteins such as podocalyxin have been found to be transported and exocytosed to the apical membrane initiation site, thereby opening and expanding the lumen [104]. Rab35 has been shown to localize to the nascent apical membrane prior to the arrival of the apically directed vesicles and to capture them by direct interaction with the cytoplasmic tail of podocalyxin, not through the typical tethering factors [105] (Fig. 4A). Such *trans* interactions also occur between Rab5 and its effector EEA1 during the homotypic fusion of early endosomes. EEA1 is associated with early endosomes via its C‐terminal PI3P‐binding domain and extends its intermediate coiled‐coil region and N‐terminal Rab5‐binding domain outward from the membrane surface, enabling the capture of other Rab5‐bearing early endosomes. Although the predicted length of EEA1 (~ 200 nm) appears to be too long to allow subsequent fusion of the two membranes, a recent study using rotary shadowing electron microscopy and optical tweezers clearly answered to this problem [106]. Intriguingly, it was shown that while EEA1 alone adopted a fully extended conformation, upon binding to active Rab5 it becomes flexible enough to collapse into smaller structures (ranging from 20 to 200 nm). This causes an equilibrium shift and generates entropic force (~ 3 pN), which pulls the two vesicles together (i.e., tethering). This finding was the first evidence that an active Rab does not just recruit its effector, but induces a conformational change in its effector upon binding (Fig. 4B). Another example is the ability of Rab itself to serve as a tethering factor. The results of an *in vitro* liposome tethering assay using purified Vps21 (yeast Rab5) and chemically defined liposomes unexpectedly showed that a homotypic interaction of Vps21 is sufficient to promote liposome tethering in the absence of any effector molecules [107] (Fig. 4C). Similarly, purified Rab5A, loaded on liposomes at physiological density (equivalent to the previously reported Rab density of a single synaptic vesicle [108]), has been shown to efficiently drive liposome tethering *in vitro* [109]. In addition, homotypic tethering by Rab1A, 3A, 4A, 6A, 7A, 9A, 11A, and 33B at higher density and efficient heterotypic tethering by Rab1A and Rab9A have been demonstrated. Understanding the physiological significance of these intrinsic tethering activities of Rabs and their possible interplay with effector‐mediated tethering await further investigation.

## Human diseases, mutant animals, and Rab knockout phenotypes

Finally, we will discuss the physiological roles of Rabs by focusing on their roles as revealed by studies on human diseases, mutant animals, and Rab‐KO phenotypes (summarized in Table 1 and Table S2). Lysosome‐related organelles (LROs) are typically nondegradative, cell‐type‐specific secretory organelles, such as the melanosomes in pigment cells, dense granules in platelets, and lamellar bodies in lung alveolar type II cells. Since these organelles play important roles in various biological phenomena (e.g., skin and hair pigmentation, blood clotting, and pulmonary surfactant secretion), defects in LRO biogenesis cause human diseases, including Hermansky–Pudlak syndrome (HPS), which is characterized by oculocutaneous albinism, a prolonged bleeding time, and often pulmonary fibrosis (reviewed in Ref. [110]). The mutated genes in the hypopigmentation *pale ear* (*ep*) mouse and *light ear* (*le*) mouse have turned out to be orthologs of the human *HPS* genes *HPS1* and *HPS4*, respectively [111, 112, 113]. HPS1 and HPS4 have subsequently been shown to heterodimerize to form the BLOC‐3 complex, which exhibits GEF activity toward Rab32 and its paralog Rab38 [21]. Furthermore, double KO of Rab32/38 in mice results in severe coat color dilution and defects in hemostasis and lung morphology [114], indicating involvement of these Rabs in LRO biogenesis. The number of dense granules in Rab32/38‐KO mouse platelets is reduced, and their morphology is abnormal.

Bacteria‐containing vacuoles, in which infectious bacteria are retained in host cells, are also thought to be a kind of LROs that serve to isolate such bacteria. Interestingly, Rab32 has been shown to be recruited to *Staphylococcus aureus*‐ or *Mycobacterium tuberculosis*‐containing vacuoles in infected macrophages [115]. Other studies have demonstrated that Rab32 is also recruited to *Salmonella* Typhi‐containing vacuoles and that Rab32‐ or HPS4‐deficient mice are more susceptible to infection by the pathogen than wild‐type mice are [116, 117]. Not surprisingly, bacteria also evolved defenses against isolation in vacuoles. *S*. Typhimurium, but not *S*. Typhi, secretes GtgE and SopD2, a protease and GAP for Rab32, respectively, into the host cell cytosol that interfere with the Rab32‐dependent defense pathway [117]. Taken together, the results of these studies suggest that Rab32/38 play roles in diverse physiological processes through LRO biogenesis. Transport of LROs, such as melanosomes and dense granules, is also known to be regulated by Rab small GTPases, specifically by the Rab27 isoform (see review Ref. [118] for details).

As with Rab32, Rab20 has also been shown to have an antibacterial function. Rab20 is recruited to *M. tuberculosis*‐containing phagosomes in macrophages, and its recruitment is enhanced by the macrophage‐activating cytokine interferon‐γ [119]. In addition, whereas overexpression of Rab20 attenuates intracellular bacterial replication, KO of Rab20 results in inability of bacteria‐containing phagosomes to become acidic and proteolytic, thereby allowing bacterial replication. Consistent with these findings, after being infected with *M. tuberculosis* the lungs of Rab20‐KO mice were found to contain larger lesion areas than in infected wild‐type mice.

Other Rab‐KO mice recently reported include the following: Rab10‐KO mice, which are characterized by early embryonic lethality [120], Rab13‐KO mice, which have a smaller spleen and lymph nodes because of impaired lymphocyte migration [121], and Rab39A‐KO mice, which exhibit reduced cross‐presentation activity by antigen‐presenting cells [122]. Moreover, The International Mouse Phenotyping Consortium (IMPC) has generated over 8000 KO mouse lines to date, and the phenotypic data for more than half of the *Rab* family genes are already available on its website (https://www.mousephenotype.org/). The data available include the lethal phenotypes of Rab2A, 5C, 21, and 40C, which have not yet been reported in the literature. The CRISPR/Cas‐mediated genome editing technology has boosted the KO mouse production rate, and the IMPC now plans to generate KO mice for all of the rest of the genes (~ 10 000) in the mouse genome within 10 years.

The recent technical advances in genome editing have also led to acceleration of KO analyses in cultured cells. In particular, genome‐wide screening using a single guide RNA (sgRNA) lentiviral library has succeeded in identifying many positive and negative regulators, including Rabs, of particular phenotypes. For example, Rab34 has been found to be a positive regulator of Hedgehog (Hh) signaling, which is sensed by primary cilia and plays an essential role in the patterning of cell differentiation during development [123]. In the absence of Rab34, primary cilia formation by both cultured cells and *in vivo* was found to be inhibited [124], and another independent study by the IMPC has shown that Rab34‐KO mice exhibit typical ciliopathy phenotypes, including polydactyly and craniofacial malformation [125]. Thus, Rab34 is essential for ciliogenesis and Hh signaling, although the molecular mechanism has yet to be determined. Other cilia‐related Rabs, including Rab8 and Rab23, have also been reported (see review Ref. [126] for details). A recent advance has been the discovery that Fuzzy and Inturned, known regulators of the planar cell polarity pathway, form a Rab23‐GEF complex [29]. Although knockdown of this complex or Rab23 inhibited ciliogenesis in cultured cells, the number and length of nodal cilia were normal in Rab23‐KO mice [127]. Rab8 localizes on primary cilia and has been shown to be required for ciliogenesis in cultured cells and a zebrafish model [128, 129], but not in mice [130]. Taken together, Rab8 and Rab23 seem not to be essential for ciliogenesis itself, but to be likely to regulate ciliary functions in mammals.

Another CRISPR screening revealed that loss of Rab10 or its chaperone RABIF (see the above section) protected cells from *L. pneumophila* toxicity by attenuating its intracellular replication [131]. Although the bacteria normally entered Rab10‐KO or RABIF‐KO cells, the *Legionella*‐containing vacuoles (LCVs) were unable to recruit the ER‐resident proteins to its membrane, indicating that *L. pneumophila* needs to hijack the host Rab10 function to convert the LCV membrane into an ER‐like membrane. Interestingly, the same as Rab33B (see the above section), Rab10 is also ubiquitinated by the bacterial proteins SidC and SdcA, and these proteins are required for Rab10 recruitment to LCVs. Both Rab10 and RABIF have also been identified as positive regulators of the surface translocation of GLUT4 [82], thereby validating the usefulness of genome‐wide screenings as a means of identifying functional partners of Rabs (e.g., GEFs, GAPs, effectors, and chaperones).

In order to facilitate more detailed and comprehensive Rab‐KO analyses, a collection of KO cell lines for the entire Rab family has been generated using the epithelial cell line Madin–Darby canine kidney (MDCK) II [132] [available from RIKEN BioResource Research Center Cell Bank (https://cell.brc.riken.jp/en); Cat#: RCB5099–RCB5148], and subsequent analyses of these cells confirmed the roles of Rab2, Rab7, and Rab11 in Golgi integrity, lysosome homeostasis, and single lumen formation in epithelial cysts, respectively. In addition, Rab6‐KO cells lack the basement membrane of polarized MDCK II cells likely due to a secretory defect. Rab6 seems to be required for the post‐Golgi transport of a wide range of secretory cargos, and cargos have been shown to be mistargeted to lysosomes for degradation in Rab6‐KO cells. Since many of the Rab‐KO phenotypes have become visible only when close paralogs, for example, Rab6A and Rab6B, were simultaneously knocked out, future research using the collection is expected to reveal new phenotypes that have been missed by genome‐wide screenings.

## Perspectives

As described above, biochemical analyses, including by mass spectrometry and proximity biotinylation, have continued to provide mechanistic insights into Rab functions by identifying novel effectors, GEFs, and GAPs, as well as post‐translational modifiers. In addition, electron microscopy techniques have been useful not only to study intracellular organelles but also for the structural analysis of purified proteins without crystallization. This allowed the study of high molecular weight Rab effectors, such as EEA1. In phenotypic studies, CRISPR/Cas technology has made it possible to accelerate KO analyses in both animals and cultured cells. Moreover, since many Rabs are involved in complicated neuronal disorders [133], pathogen defense pathways [134], and cancer [135], specific assays beyond the standard phenotyping pipeline are also needed. Such efforts will together provide a more detailed and comprehensive understanding of the physiological functions of the Rab family of GTPases in the future.

## Conflict of interest

The authors declare no conflict of interest.

## Author contributions

YH, SH, and MF prepared the figures and tables. YH and MF wrote and edited the paper.

## Supporting information


**Fig. S1.** Amino acid sequences of Rabs/Ypts that were used for the phylogenetic analysis in Fig. 2A.
**Fig. S2.** Amino acid sequences of the switch II region of human Rabs that were used for the phylogenetic analysis in Fig. 2B.
**Table S1.** Rab effectors, binding molecules, GEFs, and GAPs in mammals
**Table S2.** Mouse and human *Rab* genes.
