# Supplementary material for: Rab family of small GTPases: an updated view on their regulation and functions
Source: FEBS J. 2020 Jul 1;288(1):36–55. doi: 10.1111/febs.15453 (PMC7818423; doi:10.1111/febs.15453)
Supplement: Supplementary file 1 — Fig. S1. Amino acid sequences of Rabs/Ypts that were used for the phylogenetic analysis in Fig. 2A. Fig. S2. Amino acid sequences of the switch II region of human Rabs that were used for the phylogenetic analysis in Fig. 2B. Table S1. Rab effectors, binding molecules, GEFs, and GAPs in mammals Table S2. Mouse and human Rab genes. [file FEBS-288-36-s001.zip › febs15453-sup-0001-Supinfo.pdf]

FEBS PRESS
science publishing by scientists
WILEY
The FEBS Journal
www.febsjournal.org

Rab family of small GTPases: an updated view on their regulation and functions

Yuta Homma, Shu Hiragi and Mitsunori Fukuda

DOI: 10.1111/febs.15453

Table S1. Rab effectors, binding molecules, GEFs, and GAPs in mammals

Name (NCBI)	Effector/Binding protein	GEF	GAP	Review
Rab1A	C9orf72 [27334615], GCC185 [18946081], Giantin [17475246], golgin-84 [12538640],GM130, GRASP65 [11285137], HACE1 [21988917], MICAL-1, -cl [12788069, 18256213], mTORC1 [25446900], OCRL1, INPP5B [16902405, 18256213], optineurin [28843006], p115/Uso1 [10903204], PLEKHM2/SKIP [21737958], RUSC2/Iporin [15796781, 21737958], TBC1D13 [22762500]	TRAPPII? [19656848, 29273580], TRAPPIII? [28536105, 29273580]	TBC1D20 [17684057, 17901050, 21680502]	[15979508]
Rab1B	Cog6 [18256213], GBF1 [17429068], GCC185 [18946081], GM130 [11306556], Internal ribosome entry site (IRES) elements [30655362], MICAL-1, -cl [18256213], Mss4 [21194374], MTMR6 [23188820], OCRL1, INPP5B [18256213, 21378754], p115/Uso1 [25332841], RUSC2/Iporin [15796781, 21737958], Sec23, Sec24, Sec31 [21093099], TRAF3 [31375559]	TRAPPIII? [28536105]	TBC1D20 [21680502]	
Rab2A	ARFGEF3 [31294692], ARFRP1 [22505585], GAPDH [19557163, 15485821], GCC185 [18946081], golgin-45, GRASP55 [11739401], ICA69/Islet cell autoantigen of 69kDa [18187231], microtubule [19106097], Noc2 [27927751], PKCι/λ [14570876], Vps39, Vps41 (HOPS complex) [28063257, 28483915, 30957628], RUBCNL/PACER, STX17 [30957628], STAMBPL1(STAM binding protein like 1) [31294692]		RUTBC1/SGSM2? [29527623], TBC1D20 [17684057], TBC1D25/OATL1 [16923123]	
Rab2B	GCC185 [18946081], Gmcl1, GARI, GARI-L1-L5 [18256213, 26209634, 28930687]			
Rab3A	α-synuclein(A30P) [15099020], calmodulin [10545100, 11879192], CD63 [28900422], DENN/Rab3 GEP [18849981], Metallothionein-3 [15736926], Mss4 [21194374], Munc18-1 [17919117], myosin II [27325790], myosin Va [21349835], myosin Vc [31698103], OCRL1, INPP5B [18256213], rabphilin, Rim1, Rim2, Noc2 [8384302, 9252191, 10748113, 11056535, 11134008, 12578829], Rabin8/3 [7532276, 27170183], Slp4/granuphilin [12058058, 12176990], SNAP-29 [19170188], synapsin I [15265865, 15265868], synaptotagmin I [24472545],	DENN/Rab3 GEP [9020086], GRAB [11516400]	Rab3A-GAP [9030515, 9733780, 1311081, 19077034]; TBC1D10B/FLJ13130 [21349835]	[9078389, 9530492, 18726178]
Rab3B	calmodulin [11741295], Gas8 [18396146], myosin Va [24006491], OCRL1, INPP5B [18256213], PI3K [11748228], polymeric Ig receptor/plgR [11832247], rabphilin, Rim1, Rim2, Noc2 [8636125, 12578829, 15003533], Rabin8/3 [7532276, 27170183]		Rab3A-GAP [9030515, 9733780]	[18726178]
Rab3C	myosin Va [24006491], OCRL1, INPP5B [18256213], rabphilin, Rim1, Rim2, Noc2 [7946335, 12578829], Rabin8/3 [27170183], Zwint-1 [18625232]	DENN/Rab3 GEP [9020086]	Rab3A-GAP [9030515, 9733780]	[18726178]

Rab3D	calmodulin [27897225], myosin Va [21080055, 24006491], OCRL1, INPP5B [18256213], polymeric Ig receptor/pIgR [18171724], Rim1, Rim2, Noc2, rabphilin [12578829, 14522985], Rabin8/3 [7532276, 27170183], Tctex-1 [21262767]	DENN/Rab3 GEP [9020086]	Rab3A-GAP [9030515, 9733780]	[12168804, 18726178]
Rab4A	α/β-catenin [17494101], angiotensin II type 1 receptor (AT_1R) [20943774], CD2AP/CMS [12559036], T-cell receptor/CD3ζ chain [19201859], D-AKAP2/AKAP10 [18256213, 19797056], dynein light intermediate chain-1 [11243854], ENaC [16389071], GRASP-1 [20098723], HACE1 [21988917], L-PGDS [31575663], NDRG1 [17786215], Nischarin [23386062], P-glycoprotein [20209493], PGD_2 DP1 receptor [31575663], Rab11-FIP1/RCP [11786538], rabaptin-4 [10698684], Rabaptin-4/5 [9524117, 10698684], Rabenosyn-5 [11788822], RABEP2 [29425100], RUFY1/Rabip4, RUFY2 [11172003, 20534812, 21737958], syntaxin 4 [11063739]	L-PGDS? [31575663]	p85α [15377662], TBC1D11/GAPCenA [17562788], TBC1D16 [23019362]	
Rab4B	AP1γ [24006255], D-AKAP2/AKAP10 [18256213], PRA1 [10329441], RUFY2 [21737958]			
Rab5A	angiotensin II type 1 receptor (AT_1R) [20943774], APPL1-2 [15016378, 17581628], $Ca_v2.3$ [22178872], Dmknδ [21423773], EEA1 [9697774], EMC6 [23182941], Exocyst complex [31221728], FHF complex [27559088], hVps39-2/TRAP1 [25750764], Huntingtin-HAP40 complex [16476778], insulin receptor tyrosine kinase substrate/IRTKS [30808710], L-, T-plastin [21426900], Mon1 [20305638], OCRL1, INPP5B, Tecpr2 [18256213, 21378754], p85/p110β [11432782, 23434372, 29170408], PI3K-C2γ [26100075], p75 neurotrophin receptor [22460790], protection of telomeres 1/POT1 [23954637], PRA1 [10329441], prostacyclin receptor [18498773], Rabankyrin-5 [15328530], Rabaptin-5, 5β [8521472, 9524117, 9524116], Rabenosyn-5 [11062261], RABEP2 [29247183], Rab5IP/Sun2 [10818110, 20070612], RN-Tre/USP6NL [15152255], RUFY1/Rabip4' [14617813], RUFY3 [20376209], Syntenin [15014045], TBCK, OSBPL9, RELCH [31294692], Vinculin [24466349], ZFYVE21 [31113953]	ALS2/alsin [12837691], ALS2-cl [15388334, 21586568], GAPVD1/GAPex-5/Rme-6 [16410077, 17189207, 18385674], Rabex-5/RabGEF1 [9323142, 15339665], Rin1-3 [11703925, 12972505, 21586568], Rin-like/Rinl [21419809, 22291991]	p85α [15377662], p120 Ras GAP [9553053], RN-Tre/USP6NL [11099046, 15152255], RUTBC3/RabGAP-5 [16086013, 16923123], SynGAP [15014045], TBC1D3/PRC17 [12359748], TSC2 [9045618]	[11208157, 11292385, 16354714, 23645161, 28620038]
Rab5B	EEA1 [10491193], OCRL1, INPP5B [18256213], Rab5B [11562778], Rab5IP/Sun2 [20070612]	Rin1 [11703925]	LRRK2 phosphorylation [25605758], RUTBC3/RabGAP-5 [16086013, 16923123]	
Rab5C	AMAP1 [22734003], EEA1 [12493736], OCRL1, INPP5B, Tecpr2 [18256213], PRA1 [10329441], TPD52 [24604726]	Rin1 [11703925]	RUTBC3/RabGAP-5 [16086013, 16923123]	

Rab6A	Bicaudal D1, D2 [2007061], BICDR-1 [20360680], Cdc42, Trio [30830239], Cog6 [18256213], DENND5A/R6IP1, 5B [17725553, 19141279, 21737958], dynactin [12401177], dynein [23783758], DYNLRB1 [18044744], ELKS/Rab6IP2A, 2B [11929610, 12391317], GCC185 [18946081], Giantin [17475246], golgins [10209123], GORAB/SCYL1BP1 [18997784, 26000619], Kif1c/Rab6-KIFL [11060022], Mint1 826 [23737971], mint3 [16207088], myosin II [20562865], myosin Va [24006491], NSF [11062069], OCRL1, INPP5B [16902405, 18256213, 21378754], Rabkinesin-6/KIF20A [9438855], RUFY2 [21737958], TMF/ARA160 [17698061], Vps13B/COH1 [25492866], Vps52 [15878329],	Ric1-Rgp1 [23091056]	GAPCenA [10202141]	[15979508, 19754447]
Rab6B	Bicaudal D1, D2 [17707369, 2007061], Cog6 [18256213], DENND5A, 5B [21737958], DYNLRB1 [18044744], ELKS/Rab6IP2A, 2B [11929610], GCC185 [18946081], myosin Va [24006491], OCRL1, INPP5B [18256213], Rabkinesin-6/KIF20A [10893188], RUFY2 [21737958]		GAPCenA? [10893188]	
Rab6C				
Rab41 (6D)	dynactin 6, syntaxin 8 [26973836]			
Rab7A (7)	angiotensin II type 1 receptor (AT_1R) [20943774], EPG5 [27588602], FYCO1 [20100911], Hook1 [15471887], missing in metastasis/MIM [30808710], myosin Vc [25324551], ORP1L/Osbpl1a [16176980, 2007061], PDZD8 [31636202], PIPKIγi5[31908013], Rab24 [27550070], Rabring7/BCA2 [12972561], Rac1 [16040606], RILP [11179213, 11696325, 22740695], Rubicon, PLEKHM1 [20943950, 20974968], SKIP [32080880], VPS34/p150 [14617358], Vps26/29/35 (retromer) [18981234], Vps39, Vps41 (HOPS complex) [28063257, 30957628], vimentin [23458836], WDR91 [28860274], XAPC7 [14998988]	Mon1-Ccz1 [20305638, 20797862, 23084991], class C VPS/HOPS complex? [16143105], Folliculin [28656962]	TBC1D2A/Armus [20116244, 26100023], TBC1D5 [19531583, 27827364, 29158324], TBC1D15 [16055087, 24569479]	[20851765, 27548222, 0333976]
Rab7B (42)	Atg4B [28835545], myosin II [25217632]		TBC1D5 [30111580]	
Rab8A	α_{2B}-AR, β_2-AR [20424170], α-synuclein [24983211], BAG6 [30804014], Cep164 [23253480], DCDC5 [22159412], Dzip1 [25860027], EHBP1L1 [26833786], Endospanin-1/2 [23772379], FIP-2/optineurin [11137014, 18413238], Klotho [31707148], LAX [24515439], Map4k2/Rab8ip [8643544], Mss4 [21194374], Myosin Va [24478457], Myosin Vb [17507647], Myosin Vc [25324551], MICAL-1, MICAL-L1, L2 [18094055, 18256213, 19864458, 27552051], MICAL3 [21596566], OCRL1, INPP5B [18256213, 21378754], optineurin [11834836], PI3Kγ [25022365], RILP-L1, RILP-L2 [29125462], Rim2, rabphilin, Noc2 [12578829], Slp1/JFC1 [17105768], Slp4/granuphilin [12176990], transportin1, CTS [27633000], VAMP-3 [26034069], VAMP7 [30404838]	C9orf72 [27494456], GRAB/Rab3IL1, Rabin8 [12221131, 20937701], retinitis pigmentosa GTPase regulator (RPGR) [20631154]	TBC1D1 [17274760], TBC1D4/AS160 [15971998] , TBC1D30 [17646400]	[21850707]

Rab8B	MICAL-1, MICAL-L1, L2 [18256213, 27552051], OCRL1, INPP5B [18256213], otoferlin [18772196], TBK-1 [22921120], TRIP8b/Pex5l [11278749]	GRAB/Rab3IL1, Rabin8 [20937701]	TBC1D1 [17274760], TBC1D4/AS160 [15971998]	
Rab9A	BLOC-3 [20048159, 26620560], GCC185 [18946081], Hook1 [15471887], HPS3 [31294692], INPP5B [18256213], Nde1 [18256213], Nischarin [23386062], RABEPK/p40 [9230071], RhoBTB3 [19490898], RUTBC1 [21808068], RUTBC2 [22637480], TIP47 [11359012], TBC1D15 [24578385], vimentin [18681838]	DENND2 [20937701]		
Rab9B	BLOC-3 [30837268], GCC185 [18946081], RUTBC2 [25220469]	DENND2 [20937701]		
Rab10	dynamin2 [31808574], EHBP1 [28028537], Evi5 [18256213], GGA2 [31076515], JIP1 [24478353], KIF13A/B [30700496], MARCKS [24662485], MICAL-1, MICAL-L1, L2 [18256213, 27552051], Mss4 [7988552, 21194374, 28894007], myosin Va-c [19008234], Lgl1 [21856246], optineurin [30945962], RILP-L1, RILP-L2 [29125462], Rim1 [12578829], Sec15 [26299925], SEC16A [27354378], TBC1D13 [22762500], TBC1D21/MGCRABGAP [28067790]	DENND4 [20937701], Rabin8 [27170183]	Evi5-like/EVI5L [16923123], TBC1D1 [30275018], TBC1D4/AS160 [15971998]	[29377137]
Rab11A	α-synuclein [25092884], ALS2 [31294692], angiotensin II type 1 receptor (AT$_1$R) [20943774], β_2-AR [18983266], D-AKAP2/AKAP10 [19797056], DENND5A/R6IP1 [17725553], Evi5 [17229837], flotillin-1/reggie-1 [23825023], Folliculin [30446510], Gβ1γ2 [18701709] GRAB/Rab3IL1 [24140058], HACE1 [21988917], Hook1 [15471887], HSP90 [19193894], KIF13A [24462287], Moesin [23376974], Munc13-4 [26637356], myosin Vb [11408590], p0071/plakophilin 4 [24163434], p50RhoGAP [16380373], PI4Kβ [14767056, 24876499], prostacyclin receptor [18832025], thromboxane A2 receptor β [16126723], PS1/2 [10369872], Rab11-FIP1-5 [11495908, 11163216, 11481332, 12470645, 11786538, 18256213], Rabin8 [18256213, 20308558], RELCH/KIAA1468 [29514919], rhodopsin [25378153], Sec15[15292201], SH3TC2 [27068304], TBC1D12 [28384198], TBC1D14 [22613832], TRPV5/6 [16354700], V-ATPase ε subunit [20717956], VAMP7 [30404838], WDR44/rab11BP[10077598, 10464283, 31204173]	DENND4A/Crag [23226104], huntingtin [18845944], SH3BP5, SH3BP5L [30217979, 30872413], TRAPPII? [29273580]	Evi5 [17099728], TBC1D9B [25232007], TBC1D11/GAPCenA [17562788], TBC1D15 [28168758]	[14532427, 23176481]
Rab11B	calcium-activated K^+ channel [22935415], cGK-II [18656450], Evi5 [17229837], GRAB/Rab3IL1 [24140058], myosin Va [24006491], myosin Vb [11408590], Rab11-FIP1/2/3/5 [11495908, 18256213], Rabin8 [27170183], RELCH/KIAA1468 [29514919], SLC12A5/KCC2 [27505893], V-ATPase ε subunit [20717956]	SH3BP5, SH3BP5L [30872413]	TBC1D8B [30661770], TBC1D9B [25232007]	[19609864, 23176481]
Rab12	Mss4 [21194374], RILP [26740112], RILP-L1 [18256213, 22740695], RILP-L2 [29125462]	DENND3 [20937701]		

Rab13	Endospanin-1/2, [23772379], GGA2 [31076515], MICAL-1, MICAL-L1, L2 [16525024, 18256213, 27552051], Mss4 [21194374], OCRL1, INPP5B [18256213], δPDE [9712853], PKA-αcatalytic subunit [16473634]	DENND1C [20937701], DENND2B [25713415]		[27044746]
Rab14	Annexin A2 [18332131], Cdc42 [27901125], D-AKAP2/AKAP10 [18256213], Endotubin, VIP/MAL [18429929], Rab11-FIP1/RCP, FIP2, FIP5/Rip11 [18256213, 19702578], KIF16B [21238925], myosin Va [24006491], Nischarin [23386062], PKCι [25694446], RUFY1/Rabip4 [20534812], UT-A1 urea transporter [23796783]	DENND6A/FAM116 [22595670]	TBC1D1 [30275018], TBC1D4/AS160 [15971998]	
Rab15	Mss4 [12105226], MICAL-1, MICAL-L1, L2 [18256213, 27552051], Munc13-4 [22899725], Osgin2 [18256213], REP15 [16195351]			
Rab17	Tecpr2 [18256213]	ALS2, Rabex-5/RabGEF1 [23430262]	TBC1D7 [17646400]	
Rab18	DFCP1 [30970241], Mss4 [21194374], NAG, RINT1, ZW10 [29367353]	Rab3GAP [24891604], TRAPP II [28003315]	TBC1D20 [26063829]	[30830238]
Rab19	D-AKAP2/AKAP10 [18256213], GCC88, Golgin-97 [19001129], Osgin2, Wdr38 [18256213]			
Rab20	INPP5E[18256213, 25269936]			
Rab21	α-integrin [16754960], APPL1 [17581628], EGFR [22525675], MACF1 [22705394], PS1 [28547526], rabphilin [28636945], TMED9/10 [31455601]	MTMR13/Sbf [22648168], Varp [16525121], Rabex-5/RabGEF1 [15339665, 23430262], Rin-like/Rinl [22291991]		
Rab22A	APPL2 [23055524], BLOC-1, BLOC-2, KIF13A [30404817], CD147 [28433697], EEA1 [11870209], KIF13A [30404817], Rabex-5 [19759177], Rabenosyn-5, TBC1D2B/mKIAA1055 [20070612, 20534488]	Rabex-5/RabGEF1 [15339665], Rin-like /Rinl [21419809]	TBC1D18 [16923123]	
Rab22B (31)	APPL2 [23055524], EEA1, EGFR [24644286], OCRL1, INPP5B, Tecpr2 [18256213, 19795375, 18256213], p75 neurotrophin receptor [22460790], TBC1D2B/mKIAA1055 [20070612]	ALS2, ALS2-cl, RIN1-3 [21586568], Rin-like/Rinl [22291991] Gapex-5//GAPex-5 [17189207]		
Rab23		Inturned–Fuzzy [31564489]	EVI5L [17646400]	
Rab24	APPL2 [23055524], CtBP1[18256213], drs [19368996], RILP, Rab7 [27550070], cyclophilin A, GABARAP [16596256],			[23176493]
Rab25	myosin Vb [11408590], Rab11-FIP1/2/3/5 [11495908], β1 integrin [17925226], RIN1 [28612496]	SH3BP5, SH3BP5L [30217979, 30872413]		[28969096]
Rab26	α_{2B}-AR [23105096], Atg16L1 [25643395], RILP [31624142], Rim1 [12578829],	Plekhg5 [29084947]	TBC1D6 [31291588]	

Rab27A	Slp1-5, Slac2-a/Mlph, Slac2-b, Slac2-c/MyRIP [11773082, 11865063, 11887186, 11964381, 11980908, 12051743, 12221080], rabphilin, Noc2 [12578829, 14722103], Munc13-4 [14699162], ATP1a1 [25051489], CaV1.3 subunit [26235199], coronin 3 [18768935], IQGAP1 [24100016]	DENN/Rab3 GEP [18559336]	TBC1D10A/EPI64 [16923811]	[17664848, 18726178, 23378593, 23678941, 25947911]
Rab27B	GCC185 [18946081], Slp1-5, Slac2-a/Mlph, Slac2-b, Slac2-c/MyRIP [11856727, 11956164, 12221080, 14699162, 15039459, 16716193, 16880209, 18477466, 27810912], rabphilin, Noc2 [12578829], Munc13-4 [14699162],	DENN/Rab3 GEP [18559336, 20937701]	TBC1D10B [23671284]	[17664848, 18726178, 23378593, 23678941, 25947911]
Rab28	PDE6D, KCNJ13 [30228185]	MTMR5, MTMR13 [20937701]	TBC1D1/4 [27929607]	
Rab29	C9orf72 [28334866], LRRK2 [23395371]			
Rab30	Cog4, Golga4[18256213], Golgins[19001129, 18946081], PI4KB[30290718]			
Rab32	AKAPs [12186851], LRRK2 [25360523], Myosin Vc [25324551], SNX6 [30640902], Varp [19403694]	BLOC-3 [23084991]	RUTBC1 [26620560]	[23176493, 23247405, 28430987]
Rab33A	Atg16L1 [22740627], RUFY2-3 [21737958]			
Rab33B	Atg5-Atg16L1 [26975471], Atg16L1, Atg16L2 [18448665, 22082872], GCC185 [18946081], GM130 [11718716], Ric1 [23091056], RUFY2 [21737958]		TBC1D25/OATL1 [21383079]	
Rab34	β3-integrin [29622794], Folliculin [27113757], Munc13-2 [19641095], RILP, RILP-L1, RILP-L2 [12475955, 18256213, 22740695]			
Rab35	ACAP2/Centβ2 [20070612], Fascin1 [19729655], Folliculin [29018350], MICAL1 [28230050], MICAL-L1 [18256213, 23572513], NDP52 [28848034], OCRL1 [18256213, 21706022], p85/PI3K [29662076], podocalyxin [27040773], Rusc2 [21737958]	DENND1A-C [20159556, 20937701], Folliculin [22977732]	TBC1D10A-C [17562788, 18450757], TBC1D13 [22762500], TBC1D24 [30154457]	[23905989, 27329675]
Rab36	GAPCenA/TBC1D11[20070612], GCC185 [18946081], JIP4 [25086062], JIP3, RILP, RILP-L1, RILP-L2, EhbpL1, Gripap1, Appbp2 [18256213, 22740695, 20070612], MICAL-1 [18256213]		RUTBC2 [22637480]	
Rab37	Atg5 [29229996], Munc13-4 [26931073], Rim1 [12578829]			
Rab38	Myosinv Vc [25324551], Varp [19403694]	BLOC-3 [23084991]	RUTBC1 [26620560]	[23247405, 28430987]
Rab39A	caspase-1 [19833722], GAPDH [20070612], PI3K [24349490], Rassf1 [23294242], UACA [23624502]	DENND5A-B [20937701]		[29648608]
Rab39B	GAPDH [20070612], myosin Va [24006491], PICK1 [25784538], Rassf1 [23294242], UACA [23624502]	C9ORF72 [27617292]	RUTBC3 [16923123]	
Rab40A	Cullin5 [26598620], D-AKAP2/AKAP10 [18256213], RME8 [20070612]			
Rab40B	D-AKAP2/AKAP10 [18256213], RME8 [20070612], Tks5 [27789576]			

Rab40C	D-AKAP2/AKAP10 [18256213], RME8 [20070612], Varp [25661869]	DAB2IP [29156729]
Rab40AL		
Rab42 (43)		
Rab43 (42)	Cog6, Golga4 [18256213], Rusc2 [21737958]	RN-tre [17562788]

The nomenclature of Rabs in this review is according to the NCBI database. The names of several Rabs in the report by Itoh *et al.* (2006) are different [16923123] (indicated in parentheses). Numbers in brackets indicate PubMed ID (PMID) in the NCBI database that reports Rab effectors, binding proteins, GEFs, and GAPs. Only representative publications are listed in this table (see also reviews on several Rabs for detail in the far right column).

Table S2. Mouse and human *Rab* genes

Name (NCBI)	Gene ID Human	Gene ID Mouse	Disease & Mutant animal	Knockout (KO) phenotype
Rab3A	5864	19339	*earlybird* [12244319]	Viable and fertile [7911226, 15269275]; Perinatal lethality of Rab3A/B/C/D quadruple KO mice; 30% reduction of Ca^{2+}-triggered synaptic release [15269275, 16436611]; Reduced spontaneous neurotransmitter release at neuromuscular junctions [16631140, 17640821]; Impaired spatial reversal learning [15078563]; Reduced size of the releasable vesicle pools in chromaffin cells [20716109]; Insulin secretory deficiency and glucose intolerance [12510060]; Increased constitutive exocytosis of α-MSH in pituitary cells [24205339]; Defective dense-core vesicle exocytosis in neurons from Rab3A/B/C/D quadruple KO mice [21670909]
Rab3B	5865	69908		Viable and fertile; Rab3B/C/D triple KO mice are also viable and fertile [15269275]; Impaired long-term depression of hippocampal inhibitory synapses and selective enhancement in reversal learning [21844341]
Rab3C	115827	67295		Viable and fertile [15269275]
Rab3D	9545	19340		Viable and fertile [15269275]; Increased size of secretory granules in exocrine pancreas and parotid gland [12192047]
Rab4B	53916	19342		Adipocyte hypertrophy and insulin resistance in T cell-specific KO mice [30566860]
Rab6A	5870	19346		Embryonic lethal; Defects in basement membrane formation in KO embryos; [26304202, 26641717]; Defects in general protein secretion [31142554]; Pigmentation defects in conditional KO mice [28607494]
Rab7A (7)	7879	19349	Charcot-Marie-Tooth type 2B [12545426]	Embryonic lethal; Defects in microautophagy in the visceral endoderm of KO embryos [22990867]; Reduced numbers of peripheral T cells and inhibition of autophagic flux in T cell-specific KO mice [3615463]; Defects in class-switching in antibody responses in B cell-specific KO mice [25740947]; Increased severity of acute pancreatitis in pancreas-specific KO mice [28588238]
Rab8A	4218	17274	Related to microvillus inclusion disease [17597763]	Die at postnatal week 4, defects in apical protein localization, and microvillus inclusion bodies [17597763, 24213529, 28596241]; Hyperlipidemia and hepatosteatosis in skeletal muscle-specific KO mice [28696211]
Rab8B	51762	235442		No obvious phenotype; Rab8A/B double KO mice die at postnatal week 3 [24213529]

Rab10	10890	19325		Embryonic lethal [25860786]; Defect in maturation of oligodendrocyte precursor cells in conditional KO mice [28132130]
Rab11A	8766	53869		Embryonic lethal [25063677, 25271168, 25527643]; Impaired metalloproteinase secretion [25271168]; Defects in apical protein localization, microvillus inclusion bodies, and inflammatory bowel phenotype in intestine-specific KO mice [25063677, 25527643, 25673875, 28596241]
Rab11B	9230	19326	Intellectual disability [29106825]	
Rab13	5872	68328		Viable; Reduced lymphocyte numbers and reduced lymophocyte trafficking [25074980]
Rab18	22931	19330	Warburg Micro syndrome [21473985]	Viable and fertile; Ocular and neurological abnormalities [24764192]
Rab20	55647	19332		Decreased formation of *Mycobacterium tuberculosis*-containing proteolytic phagosomes in macrophages [28494243]
Rab23	51715	19335	Carpenter syndrome [17503333], *open brain* [11449277]	Embryonic lethal; Neural-tube defects [11449277]
Rab24	53917	19336	Canine hereditary ataxia [24516392]	
Rab25	57111	53868		Increased tumor formation [20197623, 31144312]; skin barrier dysfunction [31564077]
Rab26	25837	328778		Decreased microvascular barrier function [29965781]
Rab27A	5873	11891	Griscelli syndrome type 2 [10835631], *ashen* [10859366]	Hypopigmentation; Immunodeficincy [11266470, 11266472, 11266474]
Rab27B	5874	80718		Secretory defects in various endocrine, exocrine, and immune cells [17384153, 17587407, 17761531]
Rab28	9364	100972	Cone-rod dystrophy [23746546]	Retina degeneration [30228185], postaxial polydactyly [32084271]
Rab29 (7L1)	8934	226422	Association with Parkinson's disease [20683486]	Renal enlargement and discoloration [27424887]
Rab32	10981	67844		Increased susceptibility to *Salmonella* Typhi [26867180]; Coat and eye pigment dilution, some enlarged lung multilamellar bodies, and prolonged bleeding in Rab32/38 DKO mice [31399401]
Rab33A	9363	19337		Decreased outgrowth of forebrain commissural axons in Rab33a/ba DKO zebrafish [30755680]
Rab33B	83452	19338	Dyggve-Melchior-Clausen syndrome/Smith-McCort dysplasia [22652534, 23042644]	
Rab34	83871	19376		Preweaning lethality; Polydactyly; Cleft-lip/palate; Ciliogenesis defect [27626380, 30301781]

Rab35	11021	77407	Somatic mutations in human tumors [26338797]	*Preweaning lethality
Rab38	23682	72433	*chocolate* [11917121], *Ruby* [15112108]	Hypopigmentation; Prolonged bleeding; Pulmonary fibrosis [30060521]; Coat and eye pigment dilution, some enlarged lung multilamellar bodies, and prolonged bleeding in Rab32/38 DKO mice [31399401]
Rab39A	54734	270160		Viable; reduced cross-presentation by dendritic cells [31821587]
Rab39B	116442	67790	X-linked mental retardation/Parkinson's disease [20159109, 25434005]	
Rab43 (42)	339122	69834	Association with a hereditary liver-colon cancer syndrome [31226964]	Viable and fertile; Reduced cross-presentation by dendritic cells [27899443]

The nomenclature of Rabs in this review is according to the NCBI database. The names of several Rabs in the report by Itoh *et al*. (2006) are different [16923123] (indicated in parentheses). Numbers in brackets indicate PubMed ID (PMID) in the NCBI database. The phenotypes of several KO mice (indicated by asterisks) are available from the following URL: https://www.mousephenotype.org/. Rab33A (Cat#: RBRC05799) and Rab33B KO mice (Cat#: RBRC05800) (indicated by doble asterisks) are also available from RIKEN BioResource Research Center (https://mus.brc.riken.jp/en/).

Figure S1. Amino acid sequences of Rabs/Ypts that were used for the phylogenetic analysis in Fig. 2A

>HsRab1A
MSSMNPEYDYLFKLLLIGDSGVGKSCLLLRFADDTYTESYISTIGVDFKIRTIELD
GKTIKLQIWDTAGQERFRTITSSYYRGAHGIIVVYDVTDQESFNNVKQWLQEID
RYASENVNKLLVGNKCDLTTKKVVDYTTAKEFADSLGIPFLETSAKNATNVEQS
FMTMAAEIKKRMGPGATAGGAEKSNVKIQSTPVKQSGGGCC
>HsRab1B
MNPEYDYLFKLLLIGDSGVGKSCLLLRFADDTYTESYISTIGVDFKIRTIELDGKT
IKLQIWDTAGQERFRTITSSYYRGAHGIIVVYDVTDQESYANVKQWLQEIDRYAS
ENVNKLLVGNKSDLTTKKVVDNTTAKEFADSLGIPFLETSAKNATNVEQAFMT
MAAEIKKRMGPGAASGGERPNLKIDSTPVKPAGGGCC
>HsRab2A
MAYAYLFKYIIIGDTGVGKSCLLLQFTDKRFQPVHDLTIGVEFGARMITIDGKQIK
LQIWDTAGQESFRSITRSYYRGAAGALLVYDITRRDTFNHLTTWLEDARQHSNS
NMVIMLIGNKSDLESRREVKKEEGEAFAREHGLIFMETSAKTASNVEEAFINTAK
EIYEKIQEGVFDINNEANGIKIGPQHAATNATHAGNQGGQQAGGGCC
>HsRab2B
MVNIDGKQIKLQIWDTAGQESFRSITRSYYRGAAGALLVYDITRRETFNHLTSW
LEDARQHSSSNMVIMLIGNKSDLESRRDVKREEGEAFAREHGLIFMETSAKTAC
NVEEAFINTAKEIYRKIQQGLFDVHNEANGIKIGPQQSISTSVGPSASQRNSRDIG
SNSGCC
>HsRab3A
MASATDSRYGQKESSDQNFDYMFKILIIGNSSVGKTSFLFRYADDSFTPAFVSTV
GIDFKVKTIYRNDKRIKLQIWDTAGQERYRTITTAYYRGAMGFILMYDITNEESF
NAVQDWSTQIKTYSWDNAQVLLVGNKCDMEDERVVSSERGRQLADHLGFEFF
EASAKDNINVKQTFERLVDVICEKMSESLDTADPAVTGAKQGPQLSDQQVPPHQ
DCAC
>HsRab3B
MASVTDGKTGVKDASDQNFDYMFKLLIIGNSSVGKTSFLFRYADDTFTPAFVST
VGIDFKVKTVYRHEKRVKLQIWDTAGQERYRTITTAYYRGAMGFILMYDITNEE
SFNAVQDWATQIKTYSWDNAQVILVGNKCDMEEERVVPTEKGQLLAEQLGFDF

FEASAKENISVRQAFERLVDAICDKMSDSLDTDPSMLGSSKNTRLSDTPPLLQQ
NCSC

>HsRab3C
MRHEAPMQMASAQDARYGQKDSSDQNFDYMFKLLIIGNSSVGKTSFLFRYADD
SFTSAFVSTVGIDFKVKTVFKNEKRIKLQIWDTAGQERYRTITTAYYRGAMGFIL
MYDITNEESFNAVQDWSTQIKTYSWDNAQVILVGNKCDMEDERVISTERGQHL
GEQLGFEFFETSAKDNINVKQTFERLVDIICDKMSESLETDPAITAAKQNTRLKET
PPPPQPNCAC

>HsRab3D
MASAGDTQAGPRDAADQNFDYMFKLLLIGNSSVGKTSFLFRYADDSFTPAFVST
VGIDFKVKTVYRHDKRIKLQIWDTAGQERYRTITTAYYRGAMGFLLMYDIANQ
ESFAAVQDWATQIKTYSWDNAQVILVGNKCDLEDERVVPAEDGRRLADDLGFE
FFEASAKENINVKQVFERLVDVICEKMNESLEPSSSSGSNGKGPAVGDAPAPQPS
SCSC

>HsRab4A
MSQTAMSETYDFLFKFLVIGNAGTGKSCLLHQFIEKKFKDDSNHTIGVEFGSKII
NVGGKYVKLQIWDTAGQERFRSVTRSYYRGAAGALLVYDITSRETYNALTNWL
TDARMLASQNIVIILCGNKKDLDADREVTFLEASRFAQENELMFLETSALTGEN
VEEAFVQCARKILNKIESGELDPERMGSGIQYGDAALRQLRSPRRAQAPNAQEC
GC

>HsRab4B
MAETYDFLFKFLVIGSAGTGKSCLLHQFIENKFKQDSNHTIGVEFGSRVVNVGG
KTVKLQIWDTAGQERFRSVTRSYYRGAAGALLVYDITSRETYNSLAAWLTDAR
TLASPNIVVILCGNKKDLDPEREVTFLEASRFAQENELMFLETSALTGENVEEAF
LKCARTILNKIDSGELDPERMGSGIQYGDASLRQLRQPRSAQAVAPQPCGC

>HsRab5A
MASRGATRPNGPNTGNKICQFKLVLLGESAVGKSSLVLRFVKGQFHEFQESTIGA
AFLTQTVCLDDTTVKFEIWDTAGQERYHSLAPMYYRGAQAAIVVYDITNEESFA
RAKNWVKELQRQASPNIVIALSGNKADLANKRAVDFQEAQSYADDNSLLFMET
SAKTSMNVNEIFMAIAKKLPKNEPQNPGANSARGRGVDLTEPTQPTRNQCCSN

>HsRab5B
MTSRSTARPNGQPQASKICQFKLVLLGESAVGKSSLVLRFVKGQFHEYQESTIGA
AFLTQSVCLDDTTVKFEIWDTAGQERYHSLAPMYYRGAQAAIVVYDITNQETFA

RAKTWVKELQRQASPSIVIALAGNKADLANKRMVEYEEAQAYADDNSLLFME
TSAKTAMNVNDLFLAIAKKLPKSEPQNLGGAAGRSRGVDLHEQSQQNKSQCCS
N
>HsRab6A
MSTGGDFGNPLRKFKLVFLGEQSVGKTSLITRFMYDSFDNTYQATIGIDFLSKTM
YLEDRTVRLQLWDTAGQERFRSLIPSYIRDSTVAVVVYDITNVNSFQQTTKWIDD
VRTERGSDVIIMLVGNKTDLADKRQVSIEEGERKAKELNVMFIETSAKAGYNVK
QLFRRVAAALPGMESTQDRSREDMIDIKLEKPQEQPVSEGGCSC
>HsRab6B
MSAGGDFGNPLRKFKLVFLGEQSVGKTSLITRFMYDSFDNTYQATIGIDFLSKT
MYLEDRTVRLQLWDTAGQERFRSLIPSYIRDSTVAVVVYDITNLNSFQQTSKWID
DVRTERGSDVIIMLVGNKTDLADKRQITIEEGEQRAKELSVMFIETSAKTGYNV
KQLFRRVASALPGMENVQEKSKEGMIDIKLDKPQEPPASEGGCSC
>HsRab7
MTSRKKVLLKVIILGDSGVGKTSLMNQYVNKKFSNQYKATIGADFLTKEVMVD
DRLVTMQIWDTAGQERFQSLGVAFYRGADCCVLVFDVTAPNTFKTLDSWRDEF
LIQASPRDPENFPFVVLGNKIDLENRQVATKRAQAWCYSKNNIPYFETSAKEAIN
VEQAFQTIARNALKQETEVELYNEFPEPIKLDKNDRAKASAESCSC
>HsRab7B(42)
MNPRKKVDLKLIIVGAIGVGKTSLLHQYVHKTFYEEYQTTLGASILSKIIILGDTT
LKLQIWDTGGQERFRSMVSTFYKGSDGCILAFDVTDLESFEALDIWRGDVLAKI
VPMEQSYPMVLLGNKIDLADRKVPQEVAQGWCREKDIPYFEVSAKNDINVVQA
FEMLASRALSRYQSILENHLTESIKLSPDQSRSRCC
>HsRab8A
MAKTYDYLFKLLLIGDSGVGKTCVLFRFSEDAFNSTFISTIGIDFKIRTIELDGKRI
KLQIWDTAGQERFRTITTAYYRGAMGIMLVYDITNEKSFDNIRNWIRNIEEHASA
DVEKMILGNKCDVNDKRQVSKERGEKLALDYGIKFMETSAKANINVENAFFTL
ARDIKAKMDKKLEGNSPQGSNQGVKITPDQQKRSSFFRCVLL
>HsRab8B
MAKTYDYLFKLLLIGDSGVGKTCLLFRFSEDAFNTTFISTIGIDFKIRTIELDGKKI
KLQIWDTAGQERFRTITTAYYRGAMGIMLVYDITNEKSFDNIKNWIRNIEEHASS
DVERMILGNKCDMNDKRQVSKERGEKLAIDYGIKFLETSAKSSANVEEAFFTL
ARDIMTKLNRKMNDSNSAGAGGPVKITENRSKKTSFFRCSLL

>HsRab9A

MAGKSSLFKVILLGDGGVGKSSLMNRYVTNKFDTQLFHTIGVEFLNKDLEVDG

HFVTMQIWDTAGQERFRSLRTPFYRGSDCCLLTFSVDDSQSFQNLSNWKKEFIY

YADVKEPESFPFVILGNKIDISERQVSTEEAQAWCRDNGDYPYFETSAKDATNVA

AAFEEAVRRVLATEDRSDHLIQTDTVNLHRKPKPSSSCC

>HsRab9B

MSGKSLLLKVILLGDGGVGKSSLMNRYVTNKFDSQAFHTIGVEFLNRDLEVDG

RFVTLQIWDTAGQERFKSLRTPFYRGADCCLLTFSVDDRQSFENLGNWQKEFIY

YADVKDPEHFPFVVLGNKVDKEDRQVTTEEAQTWCMENGDYPYLETSAKDDT

NVTVAFEEAVRQVLAVEEQLEHCMLGHTIDLNSGSKAGSSCC

>HsRab10

MAKKTYDLLFKLLLIGDSGVGKTCVLFRFSDDAFNTTFISTIGIDFKIKTVELQG

KKIKLQIWDTAGQERFHTITTSYYRGAMGIMLVYDITNGKSFENISKWLRNIDEH

ANEDVERMLLGNKCDMDDKRVVPKGKGEQIAREHGIRFFETSAKANINIEKAFL

TLAEDILRKTPVKEPNSENVDISSGGGVTGWKSKCC

>HsRab11A

MGTRDDEYDYLFKVVLIGDSGVGKSNLLSRFTRNEFNLESKSTIGVEFATRSIQV

DGKTIKAQIWDTAGQERYRAITSAYYRGAVGALLVYDIAKHLTYENVERWLKE

LRDHADSNIVIMLVGNKSDLRHLRAVPTDEARAFAEKNGLSFIETSALDSTNVEA

AFQTILTEIYRIVSQKQMSDRRENDMSPSNNVVPIHVPPTTENKPKVQCCQNI

>HsRab11B

MGTRDDEYDYLFKVVLIGDSGVGKSNLLSRFTRNEFNLESKSTIGVEFATRSIQV

DGKTIKAQIWDTAGQERYRAITSAYYRGAVGALLVYDIAKHLTYENVERWLKE

LRDHADSNIVIMLVGNKSDLRHLRAVPTDEARAFAEKNNLSFIETSALDSTNVEE

AFKNILTEIYRIVSQKQIADRAAHDESPGNNVVDISVPPTTDGQKPNKLQCCQNL

>HsRab12

MDPGAALQRRAGGGGGLGAGSPALSGGQGRRRKQPPRPADFKLQVIIIGSRGV

GKTSLMERFTDDTFCEACKSTVGVDFKIKTVELRGKKIRLQIWDTAGQERFNSIT

SAYYRSAKGIILVYDITKKETFDDLPKWMKMIDKYASEDAELLLVGNKLDCETD

REITRQQGEKFAQQITGMRFCEASAKDNFNVDEIFLKLVDDILKKMPLDILRNEL

SNSILSLQPEPEIPPELPPPRPHVRCC

>HsRab13

MAKAYDHLFKLLLIGDSGVGKTCLIIRFAEDNFNNTYISTIGIDFKIRTVDIEGKKI

KLQVWDTAGQERFKTITTAYYRGAMGIILVYDITDEKSFENIQNWMKSIKENAS
AGVERLLLGNKCDMEAKRKVQKEQADKLAREHGIRFFETSAKSSMNVDEAFSS
LARDILLKSGGRRSGNGNKPPSTDLKTCDKKNTNKCSLG
>HsRab14
MATAPYNYSYIFKYIIIGDMGVGKSCLLHQFTEKKFMADCPHTIGVEFGTRIIEVS
GQKIKLQIWDTAGQERFRAVTRSYYRGAAGALMVYDITRRSTYNHLSSWLTDA
RNLTNPNTVIILIGNKADLEAQRDVTYEEAKQFAEENGLLFLEASAKTGENVED
AFLEAAKKIYQNIQDGSLDLNAAESGVQHKPSAPQGGRLTSEPQPQREGCGC
>HsRab15
MAKQYDVLFRLLLIGDSGVGKTCLLCRFTDNEFHSSHISTIGVDFKMKTIEVDGI
KVRIQIWDTAGQERYQTITKQYYRRAQGIFLVYDISSERSYQHIMKWVSDVDEV
GDATSLPGCGEGASPGKARRGPDGKANASRKLCLPQPWMKTSGTHQKASRRS
LLGIRLMRSRNGRWEESKGSSWRRSMAWTSMKQVPAPTSTLKSHSRV
>HsRab17
MAQAHRTPQPRAAPSQPRVFKLVLLGSGSVGKSSLALRYVKNDFKSILPTVGCA
FFTKVVDVGATSLKLEIWDTAGQEKYHSVCHLYFRGANAALLVYDITRKDSFLK
AQQWLKDLEEELHPGEVLVMLVGNKTDLSQEREVTFQEGKEFADSQKLLFMET
SAKLNHQVSEVFNTVAQELLQRSDEEGQALRGDAAVALNKGPARQAKCCAH
>HsRab18
MDEDVLTTLKILIIGESGVGKSSLLLRFTDDTFDPELAATIGVDFKVKTISVDGNK
AKLAIWDTAGQERFRTLTPSYYRGAQGVILVYDVTRRDTFVKLDNWLNELETY
CTRNDIVNMLVGNKIDKENREVDRNEGLKFARKHSMLFIEASAKTCDGVQCAF
EELVEKIIQTPGLWESENQNKGVKLSHREEGQGGGACGGYCSVL
>HsRab19
MHFSSSARAADENFDYLFKIILIGDSNVGKTCVVQHFKSGVYTETQQNTIGVDF
TVRSLDIDGKKVKMQVWDTAGQERFRTITQSYYRSAHAAIIAYDLTRRSTFESIP
HWIHEIEKYGAANVVIMLIGNKCDLWEKRHVLFEDACTLAEKYGLLAVLETSA
KESKNIEEVFVLMAKELIARNSLHLYGESALNGLPLDSSPVLMAQGPSEKTHCT
C
>HsRab20
MRKPDSKIVLLGDMNVGKTSLLQRYMERRFPDTVSTVGGAFYLKQWRSYNISI
WDTAGREQFHGLGSMYCRGAAAIILTYDVNHRQSLVELEDRFLGLTDTASKDC
LFAIVGNKVDLTEEGALAGQEKEECSPNMDAGDRVSPRAPKQVQLEDAVALYK

KILKYKMLDEQDVPAAEQMCFETSAKTGYNVDLLFETLFDLVVPMILQQRAER
PSHTVDISSHKPPKRTRSGCCA

>HsRab21

MAAAGGGGGGAAAAGRAYSFKVVLLGEGCVGKTSLVLRYCENKFNDKHITTL
QASFLTKKLNIGGKRVNLAIWDTAGQERFHALGPIYYRDSNGAILVYDITDEDSF
QKVKNWVKELRKMLGNEICLCIVGNKIDLEKERHVSIQEAESYAESVGAKHYH
TSAKQNKGIEELFLDLCKRMIETAQVDERAKGNGSSQPGTARRGVQIIDDEPQA
QTSGGGCCSSG

>HsRab22A

MALRELKVCLLGDTGVGKSSIVWRFVEDSFDPNINPTIGASFMTKTVQYQNELH
KFLIWDTAGQERFRALAPMYYRGSAAAIIVYDITKEETFSTLKNWVKELRQHGP
PNIVVAIAGNKCDLIDVREVMERDAKDYADSIHAIFVETSAKNAININELFIEISRR
IPSTDANLPSGGKGFKLRRQPSEPKRSCC

>HsRab22B(31)

MMAIRELKVCLLGDTGVGKSSIVCRFVQDHFDHNISPTIGASFMTKTVPCGNEL
HKFLIWDTAGQERFHSLAPMYYRGSAAAVIVYDITKQDSFYTLKKWVKELKEH
GPENIVMAIAGNKCDLSDIREVPLKDAKEYAESIGAIVVETSAKNAINIEELFQGI
SRQIPPLDPHENGNNGTIKVEKPTMQASRRCC

>HsRab23

MLEEDMEVAIKMVVVGNGAVGKSSMIQRYCKGIFTKDYKKTIGVDFLERQIQV
NDEDVRLMLWDTAGQEEFDAITKAYYRGAQACVLVFSTTDRESFEAVSSWREK
VVAEVGDIPTVLVQNKIDLLDDSCIKNEEAEALAKRLKLRFYRTSVKEDLNVNE
VFKYLAEKYLQKLKQQIAEDPELTHSSSNKIGVFNTSGGSHSGQNSGTLNGGDV
INLRPNKQRTKKNRNPFSSCSIP

>HsRab24

MSGQRVDVKVVMLGKEYVGKTSLVERYVHDRFLVGPYQNTIGAAFVAKVMSV
GDRTVTLGIWDTAGSERYEAMSRIYYRGAKAAIVCYDLTDSSSFERAKFWVKE
LRSLEEGCQIYLCGTKSDLLEEDRRRRRVDFHDVQDYADNIKAQLFETSSKTGQ
SVDELFQKVAEDYVSVAAFQVMTEDKGVDLGQKPNPYFYSCCHH

>HsRab25

MGNGTEEDYNFVFKVVLIGESGVGKTNLLSRFTRNEFSHDSRTTIGVEFSTRTV
MLGTAAVKAQIWDTAGLERYRAITSAYYRGAVGALLVFDLTKHQTYAVVERWL
KELYDHAEATIVVMLVGNKSDLSQAREVPTEEARMFAENNGLLFLETSALDSTN

VELAFETVLKEIFAKVSKQRQNSIRTNAITLGSAQAGQEPGPGEKRACCISL
>HsRab26
MSRKKTPKSKGASTPAASTLPTANGARPARSGTALSGPDAPPNGPLQPGRPSLG
GGVDFYDVAFKVMLVGDSGVGKTCLLVRFKDGAFLAGTFISTVGIDFRNKVLD
VDGVKVKLQMWDTAGQERFRSVTHAYYRDAHALLLLYDVTNKASFDNIQAWL
TEIHEYAQHDVALMLLGNKVDSAHERVVKREDGEKLAKEYGLPFMETSAKTGL
NVDLAFTAIAKELKQRSMKAPSEPRFRLHDYVKREGRGASCCRP
>HsRab27A
MSDGDYDYLIKFLALGDSGVGKTSVLYQYTDGKFNSKFITTVGIDFREKRVVYR
ASGPDGATGRGQRIHLQLWDTAGQERFRSLTTAFFRDAMGFLLLFDLTNEQSFL
NVRNWISQLQMHAYCENPDIVLCGNKSDLEDQRVVKEEEAIALAEKYGIPYFET
SAANGTNISQAIEMLLDLIMKRMERCVDKSWIPEGVVRSNGHASTDQLSEEKE
KGACGC
>HsRab27B
MTDGDYDYLIKLLALGDSGVGKTTFLYRYTDNKFNPKFITTVGIDFREKRVVYN
AQGPNGSSGKAFKVHLQLWDTAGQERFRSLTTAFFRDAMGFLLMFDLTSQQSF
LNVRNWMSQLQANAYCENPDIVLIGNKADLPDQREVNERQARELADKYGIPYF
ETSAATGQNVEKAVETLLDLIMKRMEQCVEKTQIPDTVNGGNSGNLDGEKPPE
KKCIC
>HsRab28
MSDSEEESQDRQLKIVVLGDGASGKTSLTTCFAQETFGKQYKQTIGLDFFLRRIT
LPGNLNVTLQIWDIGGQTIGGKMLDKYIYGAQGVLLVYDITNYQSFENLEDWY
TVVKKVSEESETQPLVALVGNKIDLEHMRTIKPEKHLRFCQENGFSSHFVSAKTG
DSVFLCFQKVAAEILGIKLNKAEIEQSQRVVKADIVNYNQEPMSRTVNPPRSSMC
AVQ
>HsRab29(7L1)
MGSRDHLFKVLVVGDAAVGKTSLVQRYSQDSFSKHYKSTVGVDFALKVLQWS
DYEIVRLQLWDIAGQERFTSMTRLYYRDASACVIMFDVTNATTFSNSQRWKQD
LDSKLTLPNGEPVPCLLLANKCDLSPWAVSRDQIDRFSKENGFTGWTETSVKEN
KNINEAMRVLIEKMMRNSTEDIMSLSTQGDYINLQTKSSSWSCC
>HsRab30
MSMEDYDFLFKIVLIGNAGVGKTCLVRRFTQGLFPPGQGATIGVDFMIKTVEIN
GEKVKLQIWDTAGQERFRSITQSYYRSANALILTYDITCEESFRCLPEWLREIEQY

ASNKVITVLVGNKIDLAERREVSQQRAEEFSEAQDMYYLETSAKESDNVEKLFL
DLACRLISEARQNTLVNNVSSPLPGEGKSISYLTCCNFN

>HsRab32

MAGGGAGDPGLGAAAAPAPETREHLFKVLVIGELGVGKTSIIKRYVHQLFSQHY
RATIGVDFALKVLNWDSRTLVRLQLWDIAGQERFGNMTRVYYKEAVGAFVVFD
ISRSSTFEAVLKWKSDLDSKVHLPNGSPIPAVLLANKCDQNKDSSQSPSQVDQFC
KEHGFAGWFETSAKDNINIEEAARFLVEKILVNHQSFPNEENDVDKIKLDQETLR
AENKSQCC

>HsRab33A

MAQPILGHGSLQPASAAGLASLELDSSLDQYVQIRIFKIIVIGDSNVGKTCLTFRF
CGGTFPDKTEATIGVDFREKTVEIEGEKIKVQVWDTAGQERFRKSMVEHYYRN
VHAVVFVYDVTKMTSFTNLKMWIQECNGHAVPPLVPKVLVGNKCDLREQIQVP
SNLALKFADAHNMLLFETSAKDPKESQNVESIFMCLACRLKAQKSLLYRDAER
QQGKVQKLEFPQEANSKTSCPC

>HsRab33B

MAEEMESSLEASFSSSGAVSGASGFLPPARSRIFKIIVIGDSNVGKTCLTYRFCAG
RFPDRTEATIGVDFRERAVEIDGERIKIQLWDTAGQERFRKSMVQHYYRNVHAV
VFVYDMTNMASFHSLPSWIEECKQHLLANDIPRILVGNKCDLRSAIQVPTDLAQ
KFADTHSMPLFETSAKNPNDNDHVEAIFMTLAHKLKSHKPLMLSQPPDNGIILK
PEPKPAMTCWC

>HsRab34

MSHLPGLELRREAPPLLGPLLSPFPLPAGSWHRQMLRSSLRFPITNSAGAPCKAA
GRMNILAPVRRDRVLAELPQCLRKEAALHGHKDFHPRVTCACQEHRTGTVGFK
ISKVIVVGDLSVGKTCLINRFCKDTFDKNYKATIGVDFEMERFEVLGIPFSLQLW
DTAGQERFKCIASTYYRGAQAIIIVFNLNDVASLEHTKQWLADALKENDPSSVL
LFLTPAQYALMEKDALQVAQEMKAEYWAVSSLTGENVREFFFRVAALTFEANVL
AELEKSGARRIGDVVRINSDDSNLYLTASKKKPTCCP

>HsRab35

MARDYDHLFKLLIIGDSGVGKSSLLLRFADNTFSGSYITTIGVDFKIRTVEINGEK
VKLQIWDTAGQERFRTITSTYYRGTHGVIVVYDVTSAESFVNVKRWLHEINQNC
DDVCRILVGNKNDDPERKVVETEDAYKFAGQMGIQLFETSAKENVNVEEMFNC
ITELVLRAKKDNLAKQQQQQNDVVKLTKNSKRKKRCC

>HsRab36

MVIAGASWMLGRAAASPTQTPPTTSTIRVARRSRVALVAMVIAAAGSGGPGRAE
PQLSQPSLDCGRMRSSLTPLGPPVSRDRVIASFPKWYTPEACLQLREHFHGQVSA
ACQRRNTGTVGLKLSKVVVVGDLYVGKTSLIHRFCKNVFDRDYKATIGVDFEIE
RFEIAGIPYSLQIWDTAGQEKFKCIASAYYRGAQVIITAFDLTDVQTLEHTRQWL
EDALRENEAGSCFIFLVGTKKDLLSGAACEQAEADAVHLAREMQAEYWSVSAK
TGENVKAFFSRVAALAFEQSVLQDLERQSSARLQVGNGDLIQMEGSPPETQESK
RPSSLGCC
>HsRab37
MWLMSEAHGAEPVLLREAARPFTQTLRLCVPSGNSKVMLLGDTGVGKTCFLIQ
FKDGAFLSGTFIATVGIDFRNKVVTVDGVRVKLQIWDTAGQERFRSVTHAYYRD
AQALLLLYDITNKSSFDNIRAWLTEIHEYAQRDVVIMLLGNKADMSSERVIRSED
GETLAREYGVPFLETSAKTGMNVELAFLAIAKELKYRAGHQADEPSFQIRDYVE
SQKKRSSCCSFM
>HsRab38
MQAPHKEHLYKLLVIGDLGVGKTSIIKRYVHQNFSSHYRATIGVDFALKVLHWD
PETVVRLQLWDIAGQERFGNMTRVYYREAMGAFIVFDVTRPATFEAVAKWKND
LDSKLSLPNGKPVSVVLLANKCDQGKDVLMNNGLKMDQFCKEHGFVGWFETS
AKENINIDEASRCLVKHILANECDLMESIEPDVVKPHLTSTKVASCSGCAKS
>HsRab39A
METIWIYQFRLIVIGDSTVGKSCLLHRFTQGRFPGLRSPACDPTVGVDFFSRLLEI
EPGKRIKLQLWDTAGQERFRSITRSYYRNSVGGFLVFDITNRRSFEHVKDWLEE
AKMYVQPFRIVFLLVGHKCDLASQRQVTREEAEKLSADCGMKYIETSAKDATN
VEESFTILTRDIYELIKKGEICIQDGWEGVKSGFVPNTVHSSEEAVKPRKECFC
>HsRab39B
MEAIWLYQFRLIVIGDSTVGKSCLIRRFTEGRFAQVSDPTVGVDFFSRLVEIEPGK
RIKLQIWDTAGQERFRSITRAYYRNSVGGLLLFDITNRRSFQNVHEWLEETKVH
VQPYQIVFVLVGHKCDLDTQRQVTRHEAEKLAAAYGMKYIETSARDAINVEKA
FTDLTRDIYELVKRGEITIQEGWEGVKSGFVPNVVHSSEEVVKSERRCLC
>HsRab40B
MSALGSPVRAYDFLLKFLLVGDSDVGKGEILASLQDGAAESPYGHPAGIDYKTT
TILLDGRRVKLQLWDTSGQGRFCTIFRSYSRGAQGVILVYDIANRWSFDGIDRWI
KEIDEHAPGVPKILVGNRLHLAFKRQVPTEQAQAYAERLGVTFFEVSPLCNFNIT
ESFTELARIVLLRHGMDRLWRPSKVLSLQDLCCRAVVSCTPVHLVDKLPLPIALR

SHLKSFSMANGLNARMMHGGSYSLTTSSTHKRSSLRKVKLVRPPQSPPKNCTR
NSCKIS
>HsRab40C
MGSQGSPVKSYDYLLKFLLVGDSDVGKGEILESLQDGAAESPYAYSNGIDYKTT
TILLDGRRVRLELWDTSGQGRFCTIFRSYSRGAQGILLVYDITNRWSFDGIDRWI
KEIDEHAPGVPRILVGNRLHLAFKRQVPTEQARAYAEKNCMTFFEVSPLCNFNVI
ESFTELSRIVLMRHGMEKIWRPNRVFSLQDLCCRAIVSCTPVHLIDKLPLPVTIKS
HLKSFSMANGMNAVMMHGRSYSLASGAGGGGSKGNSLKRSKSIRPPQSPPQNC
SRSNCKIS
>HsRab42(43)
MEAEGCRYQFRVALLGDAAVGKTSLLRSYVAGAPGAPEPEPEPEPTVGAECYRR
ALQLRAGPRVKLQLWDTAGHERFRCITRSFYRNVVGVLLVFDVTNRKSFEHIQD
WHQEVMATQGPDKVIFLLVGHKSDLQSTRCVSAQEAEELAASLGMAFVETSVK
NNCNVDLAFDTLADAIQQALQQGDIKLEEGWGGVRLIHKTQIPRSPSRKQHSGP
CQC
>HsRab43(41)
MAGPGPGPGDPDEQYDFLFKLVLVGDASVGKTCVVQRFKTGAFSERQGSTIGV
DFTMKTLEIQGKRVKLQIWDTAGQERFRTITQSYYRSANGAILAYDITKRSSFLS
VPHWIEDVRKYAGSNIVQLLIGNKSDLSELREVSLAEAQSLAEHYDILCAIETSA
KDSSNVEEAFLRVATELIMRHGGPLFSEKSPDHIQLNSKDIGEGWGCGC
>CeRab1
MAAMNPEYDYLFKLLLIGDSGVGKSCLLLRFADDTYTESYISTIGVDFKIRTIEL
DGKTIKLQIWDTAGQERFRTITSSYYRGAHGIIVVYDITDQETFNNVKQWLQEID
RYACENVNKLLVGNKCDLTAKRAVETQAAQDYAGQLGIPFLETSAKSSTNVEQ
AFLTMASEIKSRMGPVQGAGGAPGVRITGSQPVQDKKSGGCC
>CeRab2
MSYAYLFKYIIIGDTGVGKSCLLLQFTDKRFQPVHDLTIGVEFGARMVTIDGKQI
KLQIWDTAGQESFRSITRSYYRGAAGALLVYDITRRDTFNHLTSWLEDARQHSN
SNMVIMLIGNKSDLEARREVKREEGEAFAREHGLVFMETSAKTAANVEEAFIDT
AKEIYRKIQEGVFDINNEANGIKLGPQHSPSSPNSPGGNATGGLGGGSGCC
>CeRab3
MAAGGQPQGATPGQPDQNFDYMFKLLIIGNSSVGKTSFLFRYCDDSFTSAFVST
VGIDFKVKTVFRGDKRVKLQIWDTAGQERYRTITTAYYRGAMGFILMYDITNEE

SFNSVQDWCTQIKTYSWENAQVVLVGNKCDMDSERVVSMDRGRQLADQLGL
EFFETSAKENINVKAVFEKLVEIICDKMAESLDKDPQQQPKGQKLEANPTQKPA
QQQCNC
>CeRab5
MAARNAGTARPGGPNRTCQFKLVLLGESAVGKSSLVLRFVKGQFHEYQESTIGA
AFLTQTVCLDDATIKFEIWDTAGQERYHSLAPMYYRGAQAAIVVYDITNQESFQ
KAKNWVKELQRQASPNIVMALAGNKADVANKRTVEYEEANAYAEDNALLFM
ETSAKTSMNVNDIFMAIAKKLPIGPAQGEPTGTVDMNQPQQQQKGSCCK
>CeRab6A
MSDFGNPLKKFKLVFLGEQSVGKTSLITRFMYDSFDNTYQATIGIDFLSKTMYLE
DRTVRLQLWDTAGQERFRSLIPSYIRDSTVAVVVYDITNSNSFHQTSKWIDDVRT
ERGSDVIIMLVGNKTDLSDKRQVTTDEGERKAKELNVMFIETSAKAGYNVKQL
FRRIAGALPGIIKDDPVEPPNVVTMDPIRQRQIVTDEGSCWC
>CeRab6B
MADFTNNALKKFKLVFLGEQSVGKTSIITRFMYDSFDNTYQATIGIDFLSKTMYL
EDRTIRLQLWDTAGQERFRSLIPSYIRDSSVAVVVYDITNANSFHQTTKWVDDVR
NERGCDVIIVLVGNKTDLADKRQVSTEDGEKKARDLNVMFIETSAKAGYNVKQ
LFRKIATALPGIVQEETPEQPNIVIMNPPKDAEESQGRQCPC
>CeRab7
MSGTRKKALLKVIILGDSGVGKTSLMNQYVNRRFSNQYKATIGADFLTRDVNID
DRTVTLQIWDTAGQERFQSLGVAFYRGADCCVLAFDVTNAASFKSLDSWRDEF
LIQASPRDPDHFPFVLLGNKVDLESQRAVSSKRAQSWCQTKGNIPYYEVSAKEA
LNVEAAFLAIARDALARESQETNDFPEFPDQIRLNPNQQNQQNSGCNC
>CeRab8
MAKTYDYLFKLLLIGDSGVGKTCVLFRFSDDSFNNSFISTIGIDFKIRTIELDGKKI
KLQIWDTAGQERFRTITTAYYRGAMGIILVYDITNERSFENIKNWIRNIEEHAASD
VERMIIGNKCDIEERREVSRDRGEQLAIEYGTKFLETSAKANLNIDEAFFTLARDI
KSKMEQNEMRAGGSVSNTGRVNVGGSGTQKKSFFSNWSCNLL
>CeRab10
MARRPYDMLFKLLLIGDSGVGKTCILYRFSDDAFNTTFISTIGIDFKIKTIELKGK
KIKLQIWDTAGQERFHTITTSYYRGAMGIMLVYDITNAKSFDNIAKWLRNIDEH
ASEDVVKMILGNKCDMSDRRVVSRERGEKIAQDHGISFHETSAKLNVHVDTAF
YDLAEAILAKMPDSTDEQSRDTVNPVQPQRQSSSGGCC

>CeRab11A

MGSRDDEYDYLFKVVLIGDSGVGKSNLLSRFTRNEFNLESKSTIGVEFATRSISV
EGKTVKAQIWDTAGQERYRAITSAYYRGAVGALLVYDIAKHVTYENVERWLKE
LRDHADQNIVIMLVGNKSDLRHLRAVPTDEAKIYAERNQLSFIETSALDSTNVEA
AFTNILTEIYKSVSNKHVGTDRQGYGGGSGTIIPSPASDPPKKQCCIP

>CeRab11B

MGNEYYYLFKIVLIGNPGVGKSNLLSRFTRNEFNLKSKPTIGVEFATKIISVEGKA
VKVQIWDTAGMERFRCGASSYYRGALGALLVYDISKHKTYESVEQWLKVLRD
HANEDIVITLVGNKSDLSHAVPTDEAKIYAERNHISFIETSALDNTNVEAAFTNIV
TEIYKLVSEKYKDHSGTIIPSTASNTPKNQCCFP

>CeRab14

MTAAPYNYSYIFKYIIIGDMGVGKSCLLHQFTEKKFMADCPHTIGVEFGTRIIEV
SGQKIKLQIWDTAGQERFRAVTRSYYRGAAGALMVYDITRRSTYNHLSSWLAD
AKSLTNPNTAIFLIGNKADLEDQRDVPYEEAKAFAEENGLTFLECSAKTGSNVED
AFLETAKQIYQNIQDGSLDLNAADTGVQPKQNLPRAAENNGKKDCNC

>CeRab18

MSDDSSSPLTTLKILIIGESGVGKSSLMLRFVDDVFDPEQAATIGVDFRVTSMAID
GNRVKLAIWDTAGQERFRTLTPSYYRGAQGVICVYDVTSRSSFEKLNHWMQEV
DTYCTNDNIIKMMVANKIDMPNRVVTREEGLKFAKRHRTLFIEASAKTKEGVQC
TFEELIEKIIQTPDLWDNDRPSFRLGQPTGSSGGGGMCGC

>CeRab19

MDNDDGFDYLFKIVLVGDMGVGKTCVVQRFRNGTFVDRQGTTIGVDFTMKTL
VVDGKRVKLQIWDTGGQERFRTITQSYYRSANGIVLCYDITCKQSFGSLQRWID
DVSKFAAPNVVKLLIGTKCDLEDQRAIEAEEAEMLQRANGMFAMLETSAKGNV
NVDNAFLELATILKRQYDQGVVEQGSSGTFQLGSGGTTALGSPWQRCCQYT

>CeRab21

MLETNVDQKSFKFKIVLLGEGCVGKSSLVLRFVENKFSCKHLSTIQASFQNKTV
NVEDCQADLHIWDTAGQEKYHALGPIYYRGSNGVLLVFDITDRKSFEKVKNWV
LEIKTCLGNTAEILIVGNKIDLEEERQVTRQDAEAYAESEGALYMETSAQDNVGI
SDAFESLTAKMIEHSRTRSTEPPSTNRSIRLIDNDEAERSKKCCR

>CeRab27

MGDYDYLIKFLALGDSGVGKTSFLHRYTDNTFTGQFISTVGIDFKEKKVVYKSS
RGGFGGRGQRVLLQLWDTAGQERFRSLTTAFFRDAMGFILIFDITNEQSFLNIRD

WLSQLKVHAYCEQPDIIICGNKADLENRRQVSTARAKQLADQLGLPYFETSACT
STNVEKSVDCLLDLVMQRIQQSVETSSLPLSECRGVSLDGDPSAASSYCANC
>CeRab28
MTTMGEDEAPALPKKSPLPEKIDEADVDDDPDDKVIKIVVVGDGASGKTSICQR
FAKESFDKSYHQTLGLDFFSRRITLPHEMQVLVQVWDIGGQSIAGEMIDKYLTG
ANIVFLVYDVTNSKSFENAVDWLSVVKKNTKSSETPVKLVLMGNKTDLEERRV
VSVEAHKNFATSNDMMPTYVSAKTGDTVFLTFRQAVAEVLNVGLSRAEVEADI
EIVQGSVIEQPKQSDASYARRSDQSRSTSVCSIT
>CeRab30
MEDYKYLFKVVLVGNAGVGKTCLVRKFTQGIFPPGQSATIGVDFMIKTVKVGN
DKIKLQIWDTAGQERFRSITQSYYRSAHAIVLVYDVSCQPSFDCLPEWLGEIESY
ANRRVLKILVGNKVDKGDEREVPERIGRDFSDVNQFDYFLETSALDATNVDQLF
EQVATRLTNDMKLTDERVHQFRADATNSSSSTGGPIKLIDRAQTQLNSCCTRQS
>CeRab32
MAALTNNDKISYKVLVIGDPGVGKTSIIRRFVHNVFSSNYKTTIGVDFALKILPV
DENTVVHLQIWDISGQDRYGVMTRVYYKDAHAAIIVLDSTRERTIEGALRWKT
DLDQKVTLADGSPVPAILLANKCDIDNKLGDDKLYDLETNNGFVGSFRTSAKES
VGIEEAFKFLANTVISTEQGGQYDVPFMNREGNVNLDDNTTHSRYDSKCC
>CeRab33
MSEHHVNIPAPQFSTYATVIDPPKHVTATHPDELTTASHPQPTHPSAPQDPSPAVPS
TPVRVPYPTAPPPIPPAPEAVTAGPKKMALAPSSTKTYKQKRTFKVIIVGNAAVG
KTCLSFRFCCGRFPEHTEATIGVDFRERSCVIENELLRVQLWDTAGQERYRQSIV
AHYYRNVNAVVFVYDVTCRESFNDLALWIKECEKHGLVGDSEVPRILIGNKCD
VECTNRVSTDEAQMFADRNNMALFETSAKLASEADHVESIFLTLLHKLQQSKP
MHVQSQDERHQKEQERLILKANETENVEEEGFCC
>CeRab35
MAGTRDYDHLFKLLIIGDSGVGKSSLLLRFADNTFSENYITTIGVDFKIRTMDIN
GQRVKLQIWDTAGQERFRTITSTYYRGTHGVVVVYDVTNGESFGNVKRWLQEI
ENNCDSVQKVLVGNKCEENERRVVLESDARNYAQSMNISFFETSAKEDKNVEP
MFTCITSLVLTAKLANPQSASKDQSRTGGVSLKDNSGSTNQKKKCKCG
>CeRab37
MFLKVMLLGDSCTGKTCLLIRYKDGAFLNNNFISTVGIDYRNKLITMGDKKVK
LQIWDTAGQERFRSVTTSYYRDADALLLVYDIANRASFENCRNWLSQIKEYGK

EAVQVTLVGNKCDLPRAVPTDEGKRLAEAYQIPFMETSAKTGFNVDRAFLGLAE
RMLKLKYGFVPGGEMADTISVADTKKPEIARCCTFN
>CeRab39
METNFIGDDYGPLFHYQYRLIVIGDSTVGKSSLLRYFTEGKMAEISDPTVGVDFY
ARMIELRPGYRVKLQLWDTAGQEKFRSITKSYYRNSVGVLAIYDTTNRESFEHV
ENWVKEAALNLGGPSPSKCVFQLVGTKSDMDSQRQVNYEEGEYFAKYHKMKF
IETSSRTGDNVNEAFHMIAQEIQNRVDDGELRPVDGWEGLKTGIMRSQSVCLSE
RSFPQNSSAGACGC
>DmRab1
MSSVNPEYDYLFKLLLIGDSGVGKSCLLLRFADDTYTESYISTIGVDFKIRTIELD
GKTIKLQIWDTAGQERFRTITSSYYRGAHGIIVVYDCTDQESFNNVKQWLEEIER
YACENVNKLLVGNKSDLTTKKVVDHTTAAEYAAQLGIPFLETSAKSATNVEQAF
MTMAAEIKNRVGPPSSATDNASKVKIDQGRPVENTKSGCC
>DmRab2
MSYAYLFKYIIIGDTGVGKSCLLLQFTDKRFQPVHDLTIGVEFGARMITIDGKQIK
LQIWDTAGQEAFRSITRSYYRGAAGALLVYDITRRETFNHLTTWLEDARQHSNS
NMVIMLIGNKSDLDSRREVKKEEGEAFAREHGLVFMETSARTAANVEEAFINTA
KEIYEKIQEGVFDINNEANGIKIGQQHSPTNPSLPGAGGAAGAANSGCC
>DmRab3
MASGGDPKWQKDAADQNFDYMFKLLIIGNSSVGKTSFLFRYADDSFTSAFVST
VGIDFKVKTVFRHDKRVKLQIWDTAGQERYRTITTAYYRGAMGFILMYDVTNE
DSFNSVQDWVTQIKTYSWDNAQVILVGNKCDMEDQRVISFERGRQLADQLGV
EFFETSAKENVNVKAVFERLVDIICDKMSESLDADPTLVGGGQKGQRLTDQPQG
TPNANCNC
>DmRab4
MSETYDYLFKFLIIGSAGSGKSCLLHHFIESKFKDDSSHTIGVEFGSRIVNVGGKS
VKLQIWDTAGQERFRSVTRSYYRGAAGALLVYDATSRDSFNALTNWLNDARTL
ASPNIVILLVGNKKDLEEARDVTFLEASTFAQENELIFLETSAKTGENVEEAFLK
CSKTILAKIETGELDPERIGSGIQYGGAALRNLQTRQRSINKPDCTCRV
>DmRab5
MATTPRSGGASGTGTAQRPNGTSQNKSCQFKLVLLGESAVGKSSLVLRFVKGQF
HEYQESTIGAAFLTQTICIEDTVVKFEIWDTAGQERYHSLAPMYYRGAQAAIVV
YDIQNQDSFQRAKTWVKELHKQASPNIVIALAGNKADLSNIRVVEFDEAKQYA

EENGLLFMETSAKTGMNVNDIFLAIAKKLPKNDGANNQGTSIRPTGTETNRPTN
NCCK
>DmRab6
MSSGDFGNPLRKFKLVFLGEQSVGKTSLITRFMYDSFDNTYQATIGIDFLSKTMY
LEDRTVRLQLWDTAGQERFRSLIPSYIRDSTVAVVVYDITNTNSFHQTSKWIDDV
RTERGSDVIIMLVGNKTDLSDKRQVSTEEGERKAKELNVMFIETSAKAGYNVK
QLFRRVAAALPGMDSTENKPSEDMQEVVLKDSPNETKDPEGGCAC
>DmRab7
MSGRKKSLLKVIILGDSSVGKTSLMNQYVNKRFSNQYKATIGADFCTKEVVVN
DRVVTMQIWDTAGQERFQSLGVAFYRGADCCVLVYDVTAPNSFKNLDSWRDE
FLIQASPRDPDHFPFVVLGNKVDLDNRQVSTRRAQQWCQSKNDIPYYETSAKE
GINVEMAFQVIAKNALELEAEAEVINDFPDQITLGSQNNRPGNPDNCQC
>DmRab8
MAKTYDYLFKLLLIGDSGVGKTCILFRFSEDAFNTTFISTIGIDFKIRTIELDNKKI
KLQIWDTAGQERFRTITTAYYRGAMGIMLVYDITQEKSFENIKNWIRNIEENASA
DVEKMLLGNKCELTDKRQVSKERGEQLAIEYGIKFMETSAKASINVEEAFLTLA
SDIKAKTEKRMEANNPPKGGHQLKPMDSRTKDSWLSRCSLL
>DmRab9
MTNMRPPQKSKLLKVVILGDGGVGKSALLTRFVANRYEENNFHTIGVEFMNKD
IVVDGERYTLQIWDTAGQERFRALRTPFYRGSDICLLCYALDDRDSLKGLGVWR
NEFLNYADVDQDKFPFIVVGNKNDIPAQKRQVSSDAVQQWCAEQKVACHIETSS
KAATNVTDAFVLGLRQWRHMECVAEAELRQHGDTIDLTRPIRLVQRRICCTGG
GGGGGGVGQDADGDDAAMHSPGKKVFGQKRNASKAPATNYRL
>DmRab10
MAKKTYDLLFKLLLIGDSGVGKTCILFRFSDDAFTSTFISTIGIDFKIKTVELRGK
KIKLQIWDTAGQERFHTITTSYYRGAMGIMLVYDITNEKSFENIVKWLRNIDEH
ANEDVEKMILGNKCDMTDKRVVNKERGEAIAREHGIRFMETSAKSNINIERAFC
ELAEAILDKTSGRESAENQERVIIDRRNQEKAPGYSKCCA
>DmRab11
MGAREDEYDYLFKVVLIGDSGVGKSNLLSRFTRNEFNLESKSTIGVEFATRSIEV
DGKTIKAQIWDTAGQERYRAITSAYYRGAVGALLVYDIAKHLTYENVERWLREL
RDHADQNIVIMLVGNKSDLRHLRSVPTDEAKLFAERNGLSFIETSALDSTNVETA
FQNILTEIYRIVSQKQIRDPPEGDVIRPSNVEPIDVKPTVTADVRKQCCQ

>DmRab14

MTAAPYNYNYIFKYIIIGDMGVGKSCLLHQFTEKKFMANCPHTIGVEFGTRIIEV
DDKKIKLQIWDTAGQERFRAVTRSYYRGAAGALMVYDITRRSTYNHLSSWLTD
TRNLTNPSTVIFLIGNKSDLESTREVTYEEAKEFADENGLMFLEASAMTGQNVE
EAFLETARKIYQNIQEGRLDLNASESGVQHRPSQPSRTSLSSEATGAKDQCSC

>DmRab18

MADRAIKLLVIGESGVGKSSLIRRFVENKFDQNHDVTIGMDFKSKVMQVDGID
YKVALWDTAGAERFRSLTPSFYRKALGAILVYDITSRDSLVKLETWLAELDSYS
DNPNIAIIVVGNKIDEERVVDREEGRKFARKHRALFIETSAKCDQFVSDVFKDVV
EKIVSSEYFNNGNASAGLDIASDRDLEASASTCYC

>DmRab19

MTARNPQTLMALPNEEHFDFLFKIVLIGDCGTGKTCIVDRFKTGNYIERHGNTIG
VDFSMKTIAVEGKQIKLQIWDTAGQERFRTITQSYYRSANGVLIVYDITKRSSFS
NLQKWIEEVRRYTASNVLIILVGNKCDLEEQREVDFEEARQMCQYIPEILFVMET
SAKENMNVEDAFRCLANELKRQHDANNVEEVPENTITLGQGKPLKSCSSSCNL
T

>DmRab21

MSSSRTRNGPTLNFKAVLLGEGCVGKTSLVLRYMEDRFNAQHLSTLQASFVSRK
MSLEDGRRAQLNIWDTAGQERFHALGPIYYRGSDGALLVYDITDRDSFQKVKS
WVRELRQMRGTEIALIIVGNKTDLEEQRAVTHDEALQYARTVGAQYVETSAKE
NEGVAELFELLTQLMLEQLSQRQPDASPLRLQNPDTDNLNNSDDSEAPDPGDPA
GQRSCCGI

>DmRab23

MRLIQTATGGAAASVLQTHSQAQYNYTSMREDDIELAIKVVIVGNGGVGKSSMI
QRYCKGIFTKDYKKTIGVDFLERQIEIDGEDVRIMLWDTAGQEEFDCITKAYYRG
AQASVLVFSTTDRASFDAIKDWKRKVENECNEIPTVIVQNKIDLIEQAVVTADEV
ETLAKLLNCRLIRTSVKEDINVASVFRYLATKCHQLMTQSYDQVAGNQQNSSHP
PYSSTPTISAFSPTFTKSSSGTIVLRPAKKGHGSSVARKRKIVLKKCGIL

>DmRab26

MASTAVGLGGGEGDPGAGGPPAGSAHPDDASSMSDDVFEDAETTQARIEELRR
RPFGDGSYNPPAAPASVSASITTTTTQQQQHHNPSHHHQSSHHQPSHHHHHHH
HSQLSLTGSHHYHDDAIMAPVQRSATGYPGYRPSREAMQMYAYGTDDYDDDY
NDGWRSYRYDEVDMHPAPSNAHQQPFDDTVNHKTILLGDSGVGKTSFLVKYN

TGEFRLGSFSATVGIALTNKVVVVDGTRVKLQIWDTAGQERFRSVTHAYYRDA
HALLLLYDVTNKTTYDNIRAWLGEIREYAQEDVVIVLIGNKADCSGSERQVKRE
DGERLGREHNVPFMETSAKTGLNVELSFTAVARQLKSRGYEHGDDGKFNVHDF
VRDNTKARSVCAQCRNM
>DmRab27
MRAAPPEPEPLQLAGSGEQFLVLGDSGVGKTCLLYQYTDGRFHTQFISTVGIDFR
EKRLLYNSRGRRHRIHLQIWDTAGQERFRSLTTAFYRDAMGFLLIFDLTSEKSFL
ETANWLSQLRTHAYSEDPDVVLCGNKCDLLQLRVVSRDQVAALCRRYRLPYIE
TSACTGANVKEAVELLVGRVMERIENAACNREFSLLLTQSRCLPNIAYGQPEDLV
RLHDRREEPCSRRNCRNC
>DmRab30
MEDYKFLFKIVLVGNAGVGKTCLVRRFTQGLFPPGQGATIGVDFMIKTVEVEGE
KIKLQIWDTAGQERFRSITQSYYRSAHALILVYDISCQPTFDCLPDWLREIQEYAN
SKVLKILVGNKTDRDDREIPTQIGEEFAKQHDMYFLETSAKEAENVERLFYEIAA
ELIGQARSKDGSSSAAAAAAQRQSEGSSIGLGSFSAKAAQSNCCGGLASGGSNS
SQGG
>DmRab32
MATGVIQSSVVSTSAGRQNICDCLEQESAGLVKALPKSKSADQLSELTCACLRC
QYLATDLQHPNTQSGHLPVEHRHRPVSSGSGSLGALSAKESPIFVRRQICFPSAE
TQLHPPLSSSQFSLRSASPAYAEPKPELDLGAIEALQLTTVMPAGVNRNHMDINR
NNGYLHNGAIMTSTSDKREHLYKILVIGELGTGKTSFIKRYVHQFFSQNYRATIG
VDFALKVLQWDANTIVRLQLWDIAGQERFGNMTRVYYKEAVGAFIVFDVTRSG
TFDCVSKWKEDLDSKVQLPDGSPIPCILLANKCDQEKQGIITQPEKMDEYVREN
GFAGWFETSAKENINIDEAARALVNKILINDKLISADLADGDKFNLSAADATGSD
AKNKCSC
>DmRab35
MARGFDHLFKLLIIGDSGVGKSSLLIRFSDDTFSGSYITTIGVDFKIRTVDIEGMR
VKLQIWDTAGQERFRTITSTYYRGTHGVIVVYDVTNGESFANVRRWLEEIQNNC
DVVKKVLVGNKNDDPDRKVVITEDAQRFAKQMDIELFETSAKDNINVENMFLS
ITRQVLDHKLRTSPNEQQKDTLHLKPNPKGSKGGKCCR
>DmRab39
MVEPIFEYQFRLILIGDSTVGKSSLLKFFTDGKFAELSDPTVGVDFFARLIEMKDG
TQIKLQLWDTAGQERFRSITKSYYRNSVGVLLVYDISNHASFEHIPLWMMEAQR

HIEPHRPVFALVGCKLDLINAGGHREVTTEEAQKFAKQHGLHFVETSARSGANV
EEAFRMVTQEVYARIRSGEYKAEDGWDGIKSGFSRPNSLDFNLVVAEPEKSSCC
>DmRab40
MGTMTKDYDYLLKVLLVGDSDVGKHEILSNLEDPSTESPFCSGNAYKTTTILLE
GKRVKLQLWDTSGQGRFCTIIRSYSRGAQGIILVYDITNKWSFDGIDRWLKEVDE
HAPGIPKVLVGNRLHLAFKRQVAAKQAETYASRNNMSCFEISPLCNFNIRESFCE
LARMALHRNGMEHIWRSNKVLSLQELCCRTIVRRTSVYAIDSLPLPPSVKSTLKS
YALTTSQCFNSLTQSSKSKNRCKTPTSSSRNSCAIA
>ScYpt1
MNSEYDYLFKLLLIGNSGVGKSCLLLRFSDDTYTNDYISTIGVDFKIKTVELDGK
TVKLQIWDTAGQERFRTITSSYYRGSHGIIIVYDVTDQESFNGVKMWLQEIDRYA
TSTVLKLLVGNKCDLKDKRVVEYDVAKEFADANKMPFLETSALDSTNVEDAFL
TMARQIKESMSQQNLNETTQKKEDKGNVNLKGQSLTNTGGGCC
>ScYtp51
MNTSVTSIKLVLLGEAAVGKSSIVLRFVSNDFAENKEPTIGAAFLTQRVTINEHTV
KFEIWDTAGQERFASLAPMYYRNAQAALVVYDVTKPQSFIKARHWVKELHEQ
ASKDIIIALVGNKIDMLQEGGERKVAREEGEKLAEEKGLLFFETSAKTGENVND
VFLGIGEKIPLKTAEEQNSASNERESNNQRVDLNAANDGTSANSACSC
>ScYpt52
MLQFKLVLLGDSSVGKSSIVHRFVKDTFDELRESTIGAAFLSQSITIHPNDGNETK
DVVIKFEIWDTAGQERYKSLAPMYYRNANAALVVYDITQEDSLQKARNWVDE
LKNKVGDDDLVIYLLGNKVDLCQETPSTETSPDSNEGGDEEQKVRAISTEEAKQ
YAQEQGLLFREVSAKTGEGVKEIFQDIGEKLYDLKKDEILSKQNRQIGGGNNGQ
VDINLQRPSTNDPTSCCS
>ScYpt53
MDKHTAAIPTLTIKVVLLGESAVGKSSIVLRFVSDDFKESKEPTIGAAFLTKRITR
DGKVIKFEIWDTAGQERFAPLAPMYYRNAQAALVVFDVTNEGSFYKAQNWVE
ELHEKVGHDIVIALVGNKMDLLNNDDENENRAMKAPAVQNLCERENLLYFEAS
AKTGENIYQIFQTLGEKVPCPEQNTRQSSTHDRTITDNQRIDLESTTVESTRETGG
CNC
>ScYpt6
MSRSGKSLTKYKIVFLGEQGVGKTSLITRFMYDTFDDHYQATIGIDFLSKTMYL
DDKTIRLQLWDTAGQERFRSLIPSYIRDSRVAIIVYDITKRKSFEYIDKWIEDVKN

ERGDENVILCIVGNKSDLSDERQISTEEGEKKAKLLGAKIFMETSTKAGYNVKA
LFKKIAKSLPEFQNSESTPLDSENANSANQNKPGVIDISTAEEQEQSACQC
>ScYpt7
MSSRKKNILKVIILGDSGVGKTSLMHRYVNDKYSQQYKATIGADFLTKEVTVDG
DKVATMQVWDTAGQERFQSLGVAFYRGADCCVLVYDVTNASSFENIKSWRDE
FLVHANVNSPETFPFVILGNKIDAEESKKIVSEKSAQELAKSLGDIPLFLTSAKNAI
NVDTAFEEIARSALQQNQADTEAFEDDYNDAINIRLDGENNSCSC
>ScYpt31
MSSEDYGYDYDLLFKIVLIGDSGVGKSNLLSRFTKNEFNMDSKSTIGVEFATRTL
EIDGKRIKAQIWDTAGQERYRAITSAYYRGAVGALIVYDISKSSSYENCNHWLSE
LRENADDNVAVGLIGNKSDLAHLRAVPTEESKTFAQENQLLFTETSALNSENVD
KAFEELINTIYQKVSKHQMDLGDSSANGNANGASAPNGPTISLTPTPNENKKAN
GNNCC
>ScYpt32
MSNEDYGYDYDYLFKIVLIGDSGVGKSNLLSRFTTDEFNIESKSTIGVEFATRTIE
VENKKIKAQIWDTAGQERYRAITSAYYRGAVGALIVYDISKSSSYENCNHWLTE
LRENADDNVAVGLIGNKSDLAHLRAVPTDEAKNFAMENQMLFTETSALNSDNV
DKAFRELIVAIFQMVSKHQVDLSGSGTNNMGSNGAPKGPTISLTPAPKEDKKKK
SSNCC
>ScSec4
MSGLRTVSASSGNGKSYDSIMKILLIGDSGVGKSCLLVRFVEDKFNPSFITTIGID
FKIKTVDINGKKVKLQLWDTAGQERFRTITTAYYRGAMGIILVYDVTDERTFTNI
KQWFKTVNEHANDEAQLLLVGNKSDMETRVVTADQGEALAKELGIPFIESSAK
NDDNVNEIFFTLAKLIQEKIDSNKLVGVGNGKEGNISINSGSGNSSKSNCC

Figure S2. Amino acid sequences of the switch II region of human Rabs that were used for the phylogenetic analysis in Fig. 2B

>HsRab1A
GQERFRTITSSYYRGAH
>HsRab1B
GQERFRTITSSYYRGAH
>HsRab2A
GQESFRSITRSYYRGAA
>HsRab2B
GQESFRSITRSYYRGAA
>HsRab3A
GQERYRTITTAYYRGAM
>HsRab3B
GQERYRTITTAYYRGAM
>HsRab3C
GQERYRTITTAYYRGAM
>HsRab3D
GQERYRTITTAYYRGAM
>HsRab4A
GQERFRSVTRSYYRGAA
>HsRab4B
GQERFRSVTRSYYRGAA
>HsRab5A
GQERYHSLAPMYYRGAQ
>HsRab5B
GQERYHSLAPMYYRGAQ
>HsRab5C
GQERYHSLAPMYYRGAQ
>HsRab6A
GQERFRSLIPSYIRDST
>HsRab6B
GQERFRSLIPSYIRDST
>HsRab7
GQERFQSLGVAFYRGAD
>HsRab7B(42)
GQERFRSMVSTFYKGSD
>HsRab8A
GQERFRTITTAYYRGAM
>HsRab8B
GQERFRTITTAYYRGAM
>HsRab9A
GQERFRSLRTPFYRGSD
>HsRab9B
GQERFKSLRTPFYRGAD
>HsRab10
GQERFHTITTSYYRGAM
>HsRab11A
GQERYRAITSAYYRGAV
>HsRab11B
GQERYRAITSAYYRGAV
>HsRab12
GQERFNSITSAYYRSAK
>HsRab13
GQERFKTITTAYYRGAM
>HsRab14
GQERFRAVTRSYYRGAA
>HsRab15
GQERYQTITKQYYRRAQ
>HsRab17
GQEKYHSVCHLYFRGAN

>HsRab18
GQERFRTLTPSYYRGAQ
>HsRab19
GQERFRTITQSYYRSAH
>HsRab20
GREQFHGLGSMYCRGAA
>HsRab21
GQERFHALGPIYYRDSN
>HsRab22A
GQERFRALAPMYYRGSA
>HsRab22B(31)
GQERFHSLAPMYYRGSA
>HsRab23
GQEEFDAITKAYYRGAQ
>HsRab24
GSERYEAMSRIYYRGAK
>HsRab25
GLERYRAITSAYYRGAV
>HsRab26
GQERFRSVTHAYYRDAH
>HsRab27A
GQERFRSLTTAFFRDAM
>HsRab27B
GQERFRSLTTAFFRDAM
>HsRab28
GQTIGGKMLDKYIYGAQ
>HsRab29(7L1)
GQERFTSMTRLYYRDAS
>HsRab30
GQERFRSITQSYYRSAN
>HsRab32
GQERFGNMTRVYYKEAL
>HsRab33A
GQERFRKSMVEHYYRNVH
>HsRab33B
GQERFRKSMVQHYYRNVH
>HsRab34
GQERFKCIASTYYRGAQ
>HsRab35
GQERFRTITSTYYRGTH
>HsRab36
GQEKFKCIASAYYRGAQ
>HsRab37
GQERFRSVTHAYYRDAQ
>HsRab38
GQERFGNMTRVYYREAM
>HsRab39A
GQERFRSITRSYYRNSV
>HsRab39B
GQERFRSITRAYYRNSV
>HsRab40B
GQGRFCTIFRSYSRGAQ
>HsRab40C
GQGRFCTIFRSYSRGAQ
>HsRab42(43)
GQECFRCITRSFYRNMV
>HsRab43(41)
GQERFRTITQSYYRSAN
